# Cryptochrome magnetoreception: Time course of photoactivation from non-equilibrium coarse-grained molecular dynamics

**DOI:** 10.1016/j.csbj.2024.11.001

**Published:** 2024-11-10

**Authors:** Jessica L. Ramsay, Fabian Schuhmann, Ilia A. Solov’yov, Daniel R. Kattnig

**Affiliations:** a Department of Physics, University of Exeter, Stocker Rd., Exeter EX4 4QL, UK; b Living Systems Institute, University of Exeter, Stocker Rd., Exeter EX4 4QD, UK; c Niels Bohr International Academy, Niels Bohr Institute, University of Copenhagen, Blegdamsvej 17, Copenhagen 2100, Denmark; d Institute of Physics, Carl von Ossietzky Universität Oldenburg, Carl-von-Ossietzky Str. 9–11, Oldenburg 26129, Germany; e Research Centre for Neurosensory Science, Carl von Ossietzky Universität Oldenburg, Carl-von-Ossietzky-Str. 9-11, Oldenburg 26129, Germany; f Center for Nanoscale Dynamics (CENAD), Carl von Ossietzky Universität Oldenburg, Ammerländer Heerstr. 114–118, Oldenburg 26129, Germany

**Keywords:** Cryptochrome, Magnetoreception, Radical pair mechanism, Protein dynamics, Coarse-grained molecular dynamics, Network model

## Abstract

Magnetoreception, the ability to sense magnetic fields, is widespread in animals but remains poorly understood. The leading model links this ability in migratory birds to the photo-activation of the protein cryptochrome. Magnetic information is thought to induce structural changes in cryptochrome via a transient radical pair intermediate. This signal transduction pathway has been the subject of previous all-atom molecular dynamics (MD) simulations, but insights were limited to short timescales and equilibrium structures. To address this, we developed a non-equilibrium coarse-grained MD simulation approach, exploring cryptochrome’s photo-reduction over 20 replicates of 20 µs each. Our results revealed significant structural changes across the protein, with an overall time constant of 3 µs. The C-terminal (CT) region responded on a timescale of 4.7 µs, followed by the EEE-motif, while the phosphate binding loop (PBL) showed slower dynamics (9 µs). Network analysis highlighted direct pathways connecting the tryptophan tetrad to the CT, and distant pathways involving the EEE and PBL regions. The CT-dynamics are significantly impacted by a rearrangement of tryptophan residues in the central electron transfer chain. Our findings underscore the importance of considering longer timescales when studying cryptochrome magnetoreception and highlight the potential of non-equilibrium coarse-grained MD simulations as a powerful tool to unravel protein photoactivation reactions.

## Graphical abstract

**Figure:**
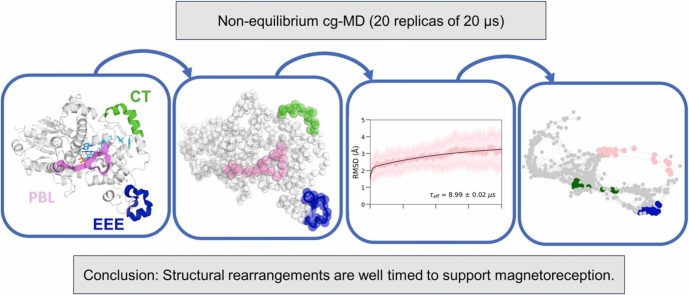

## 1 Introduction

The ability to detect magnetic fields has been reported in various animals. The geomagnetic field, with intensities ranging from 25 to 65 μT, provides a pervasive reference frame to aid navigation, from annual migrations, first demonstrated in migratory birds [1], [2], [3], to more localized journeys reported in butterflies [4] and pigeons [5], [6], [7], and even in nest building and nest finding observed in wood mice [8] and ants [9], [10]. In many species, a light-dependent, often vision-associated, compass sense has been documented. While many details of its mechanistic underpinning have remained elusive, the leading hypothesis is based on a radical pair model, originally theorized by Schulten *et al.* [11] and later further developed by Ritz *et al.* [12]. The central element of the hypothesis is a spin-correlated radical pair, which can coherently interconvert between electronic singlet or triplet states. The interconversion of these states is driven by magnetic interactions, predominantly hyperfine interactions, and can be influenced by even weak applied magnetic fields if the involved superposition states are sufficiently long-lived (on the order of 700 ns for a 50 µT magnetic field [13]). In this way, yields of subsequent spin-selective reactions are rendered magnetosensitive, enabling a compass sense when the radical pairs are sufficiently ordered and immobilized [14], [15]. The only currently known proteins that fulfil the requirements of a magnetosensor are blue-light-sensitive cryptochromes (Cry), i.e. cryptochromes that bind the flavin adenine dinucleotide (FAD) cofactor. Besides their hypothesized role in magnetoreception, such cryptochromes are essential for diverse biophysical functions, such as circadian timing [16], [17], plant growth [18] and flowering [19], [20].

Magnetoreception mediated by cryptochrome is particularly well-researched in migratory songbirds, where three different types of cryptochromes are expressed as six isoforms in the avian retina. Among these, Cry4a emerges as the leading candidate for functioning as the magnetoreceptor, exhibiting significantly higher expression levels during the migratory season and lacking circadian rhythmicity [21]. Recent experiments with ErCry4a have demonstrated sensitivity to moderate-strength magnetic fields [22], [23], although directional effects and sensitivity to the geomagnetic field have yet to be shown. Cry1a has also been discussed as a potential magnetoreceptor. However, it does not seem to bind FAD strongly in vitro [24], [25], [26], [27], exhibits daily rhythmicity [21], and interacts with clock proteins [28], which are essential for the circadian rhythm. Thus, currently the evidence supporting Cry1a's role in magnetoreception appears less compelling than that for Cry4a.

**Fig. 1:**
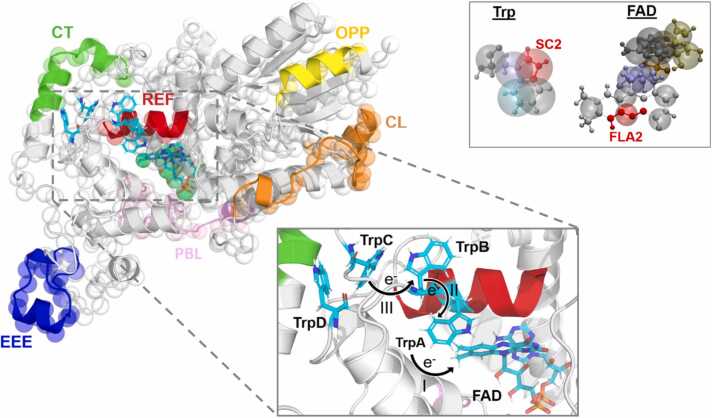
The protein structure showing both the all-atom (cartoon ribbons) and coarse-grained (cg) model (transparent spheres) of only the backbone beads overlayed, with specific regions of the protein highlighted. PBL: Phosphate Binding Loop (denoted pink); CT: C-terminal tail (green); EEE: Residues 440–460, which contain a PDZ-binding motif (blue); OPP: residues 80–90, corresponding to the surface area opposite of the CT and tryptophan tetrad (yellow); CL: Connecting Loop Residues 170–200 (orange), REF: reference α-helix residues 389–399 (red). The zoomed in image highlights the FAD and tryptophan tetrad, with the electron transfer steps leading up to the formation of FAD^•–^/Trp_C_^•+^ radical pair. The panel in the top right, illustrates the coarse-graining of the Trp and FAD residues. The distance of the tryptophan and flavin co-factor are measured as the distance of the SC2 and FLA2 beads, here highlighted in red. These beads closely correspond to the centres of the spin densities of the radicals. The flavin was parametrized following Sousa et al. [43].

Many details of the radical pair mechanism of magnetoreception are still unknown, even in relation to basic aspects. Besides the question of the relevant isoform, there is an ongoing debate about whether the magnetically sensitive radical pairs are formed directly by photoexcitation of the protein or via its re-oxidation [29], [30], [31], [32], [33], [34], [35], [36], [37], [38]. Here, we will focus on the photo-reduction of Cry4a, where upon exposure to blue light, FAD is excited (FAD*), leading to a series of electron transfers via the conserved tryptophan tetrad [39], [40], forming a radical pair comprising of FAD and the third tryptophan along the electron transfer chain, Trp_C_ (see Fig. 1). This radical pair is a transient species, which will give rise to either recombination or better separated radical pairs involving Trp_D_, the fourth tryptophan of the electron transfer chain, and potentially a surface-exposed tyrosine [41]. It is also currently unclear whether Trp_C_, or Trp_C_ and Trp_D_, interconverted by a fast degenerate electron transfer reaction [23], [42], is the crucial entity involved in the radical pair mechanism in vivo. A recent theoretical study suggested that the Trp_C_-radical-pair (RPC) is more magnetically sensitive than its Trp_D_-counterpart, suggesting that Trp_C_ could be primarily responsible for magnetic sensing and Trp_D_ better placed to initiate magnetic signaling [23]. Here, our focus is on the radical pair formed with the third tryptophan, namely Trp_C_, to investigate the initial activation steps that follow light absorption on the timescale of microseconds, i.e. times for which the magnetic field is imprinted on the radical pair’s spin dynamics and the protein starts its non-equilibrium structural rearrangements that facilitate down-stream signaling.

The current understanding of the signal transduction in cryptochrome is believed to occur via a conformational change within the C-terminal tail (CT), shown in several studies, including plants (AtCry) [44], [45], drosophila (DmCry) [46], [47] and pigeon Cry4 (ClCry4) [27], [41], [48], which are all further discussed in a recent review paper [49]. The photo-activation of DmCry, which has been studied in more detail, involves the release of the C-terminal tail [50], [51], also shown in AtCry [52], [53]. On the other hand, for Cry4 in chicken, the C-terminal region was found to become less solvent exposed upon photoexcitation [54], [55]. This was further shown in a proteolysis assays of pigeon ClCry4, where both the phosphate-binding loop (PBL) and the CT appear to be mutually protected upon activation [41]. A comparison of different avian Cry4 structures, including non-migratory and migratory species, did not exhibit significant differences but instead showed flexibility across similar regions, suggesting they may share similar activation and signaling mechanisms [50], [56], [51], [57]. In any case, the restructuring of the protein’s C-terminal tail appears to be a common feature of many cryptochromes. It has been noted in *Drosophila* that the region ranging from residues 440–460, contains a PDZ domain-binding motif, consisting of three consecutive glutamic acids (EEE), which could facilitate the formation of protein complexes and transduce the signal [46]. PDZ proteins can function as a modular scaffold to help organize and direct signaling partners to promote proximity and ensure rapid and specific signal transduction [58], [59]. Little else is known that assists and facilitates the signaling state within the protein. Thus, the goal of understanding how certain species detect magnetic field information and transduce it into neuronal impulse signals for interpretation by the brain mostly remains elusive.

Several studies have aimed to infer the early stages of Cry4 activation from equilibrium molecular dynamics (MD) simulations. In agreement with experimental findings, these studies present hints of the release or restructuring of the CT [50], [60], [61], [62]. Studies on bird cryptochromes have been undertaken for ClCry4, based on the available crystal structure, and ErCry4, based on a homology model, both with and without the truncated CT [57]. An extensive study of the former suggested that upon photo-activation, the PBL of ClCry4 swings open to allow solvents and possibly reactants access to the buried FAD in response to its photo-reduction [63]. This feature has also been recently confirmed by a follow-up coarse-grained MD study, further showing the phosphate-binding loop re-closing in the re-oxidation cycle. Thus, the PBL appears to act like a gate which is opened upon light activation and closed upon re-oxidation [64].

Employing equilibrium MD simulations allows identifying conformational features of the protein depending on its redox state. However, these simulations cannot capture the transient states and transitions crucial for understanding protein behaviour immediately after photoexcitation. Non-equilibrium MD offers the ability to capture these structural responses of proteins to perturbations, which could provide new insights into the signal transduction across the protein.

In this study, we explore the timescales of structural rearrangements during the initial phase of cryptochrome’s photo-reduction in the expectation that this will inform subsequent downstream signaling events. We study Cry4 from *C. livia*, ClCry4, as it is the only avian cryptochrome for which a crystal structure has been resolved so far [41]. We employ a coarse-grained approach to study the conformation changes following the photo-activation on the 10 µs-timescale. The FAD and tryptophan, Trp_C_, are simulated in the radical ion pair state, generated by a sudden charge shift initiated from various configurations of the equilibrated dark state. This approach allows us to study the effects of the radical-pair (RPC) generation on the overall structure of the cryptochrome protein and elucidate the timescales associated with characteristic rearrangements. Thus, this paper presents the results of coarse-grained MD simulations investigating the early-stage dynamics of bird cryptochrome photoreduction. We use 20 replicas of 20 µs, which vastly exceeds previous studies [60] and permits a more completely sampling of the initial stages of the signaling process.

## 2 Methods

### 2.1 Molecular Dynamics

A model of ClCry4 [41] was built using the Martinize2 program [65], [66], [67], [68] with the Martini22p force field. The model was equilibrated in the dark-state configuration, i.e. assuming the radical-sites in their precursor form as FAD/Trp_C_ at 310 K. The original structure was taken from an all-atom MD simulation conducted in an earlier study [63]. From a trajectory of the equilibrated system, 20 snapshots were extracted interspersed by 1 µs, and transferred to the correlated radical pair state by altering the FLA2 bead in FAD and the SC2 bead in tryptophan (Trp_C_) to reflect the different charges associated with the FAD^•–^/Trp_C_^•+^ state, and propagated without interruption, i.e. by extending the original trajectory, for 20 µs each, in the canonical ensemble.

Thus, the trajectories analysed here covered 400 µs of the FAD^•–^/Trp_C_^•+^ radical pair state. A detailed description of the model and simulation approach can be found in the earlier study [63]. The model employed GōMartini [69] virtual sites interacting through nonbonded Lennard-Jones interactions with a well depth of ε = 9.414 kJ/mol. All parameters were as in [63], [43]. The coarse-grained dark state (DS) model featured the phosphate binding loop in the closed configuration. The simulations were carried out using GROMACS [70], [71], [72], [73], [74] with temperature set to 310 K. The simulation timestep was 20 fs. All trajectories were unwrapped in regard to their periodic boundary condition using the GROMACS tool “trajconv” [71]. In this classical MD simulation, Earth’s weak magnetic field has no impact, as it influences the spin of electrons on the quantum level. The change in charge serves as an approximation of the radical pair state to simulate conformational responses of the protein to the blue-light activation. A simulation of the spin dynamics, which conversely are sensitive to the protein structural dynamics, is conducted, for instance, by Grüning et al. [42], [75].

### 2.2 Stability

The software MDTrajectories [76], [77] was used to conduct a root-mean-square-deviation (RMSD) analysis to assess the stability of the protein simulations. The protein structures were aligned to their initial frame in each trajectory. The RMSD was computed for the entire protein and for eight regions across all structures (see Fig. 1). These eight regions including the phosphate binding loop (PBL; residues 220–245), the C-terminal region (CT; residues 470–497), the connecting loop (CL; residues 170–200), the region opposite Trp_D_ (OPP; residues 80–90), the reference region (REF; residues 388–399), the region containing three consecutive glutamic acids (EEE; residues 440–460), and two unnamed regions (residues 20–33 and 110–125).

### 2.3 Trajectory Comparison

To compare the conformational changes in the ClCry4 protein and to pinpoint some of its versatile regions, the symmetrized Kullback-Leibler divergence (KLD) [78] between the distributions of positions of each amino acid residue in the RPC and resting state was employed. The KLD measure was calculated using the software package SiMBols [79].

### 2.4 Distance Analyses

The distances between the FAD cofactor, specifically from the centre of mass of the isoalloxazine ring (FLA1–5) to the centre of mass of the four individual tryptophan residues involved in the electron transfer tetrad, see Fig. 1, were extracted. These distances directly influence the electron transfer rates, which are essential for the protein’s functionality [22]. Hanić *et al.* [57] reported corresponding distances employing the centre of mass of the flavin and tryptophan residue in all-atom MD simulations. For our coarse-grained simulations, we similarly calculated distances using the centre of mass of flavin (FLA1-FLA5 beads) and tryptophans. Additionally, we measured distances between the FLA2 bead on FAD and the SC2 bead on Trp_C_ and Trp_D_, which more closely correspond to the centres of spin density of the radical pairs.

### 2.5 Network Analysis

We conducted a network analysis of the structural changes in the photo-excited protein relative to the dark state. To this end, a correlation matrix was constructed from the combined, per-residue KLD data of the 20 trajectories. The correlation matrix was binarized using a suitable threshold and the resulting matrix used as adjacency matrix to define a graph representation of correlated structural rearrangements. Subsequently, we employed the NetworkX Python package [80] to visualize and analyse the resulting correlation network. A spring layout was used to represent the network.

## 3 Results

**Fig. 2:**
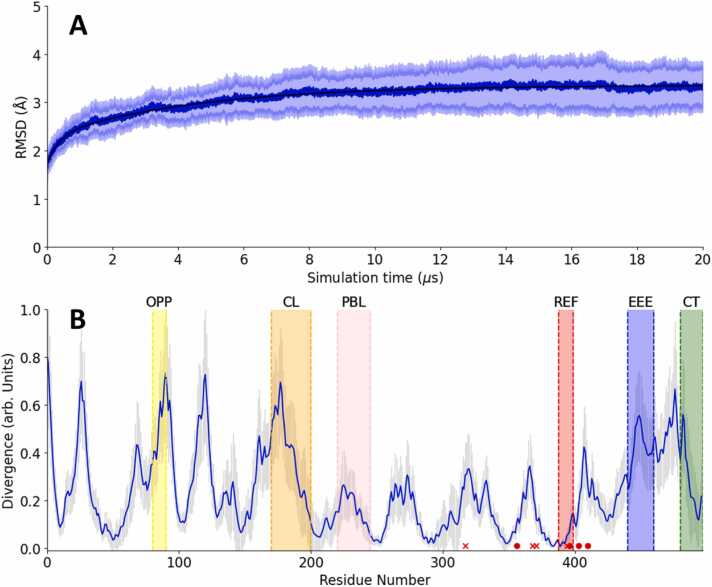
(A) Time evolution of the average RMSD of the protein backbone with standard deviation over 20 trajectories aligned to the dark state (DS). The mean RMSD levels off at 3.35 Å with an effective time constant of 3.0 µs, indicating a quasi-stable structure. The final standard deviation of the RMSD resulted in a value of 0.4 Å. The black line is a bi-exponential fit to the data. Details of the fitting function and parameters can be found in Table S1 of the SI, along with individual trajectories compiled in Fig. S1. (B) Average KLD of the coarse-grained RPC trajectories relative to the DS equilibrium simulation from an earlier study [63], with standard deviation shown as grey sticks. Significant motion is observed around residues 440–460 (highlighted in blue, denoted EEE), which contains a linear EEE motif. Other regions exhibiting clear motion include residues 170–200 (highlighted in orange; denoted CL) and 80–90 (yellow; OPP), with the latter located roughly opposite the surface exposed tryptophan, Trp_D_. Both the phosphate-binding loop (pink; PBL) and CT (green; CT) have much less motion than other regions of the protein. Additional regions spanning residues 20–33, 110–125 and 300–320 are noteworthy. Finally, residues 388–399 (red; REF) are part of an α-helix adjacent the FAD, with very little variation in this region. The red crosses indicate the four tryptophan residues which are involved in the photo-induced charge separation of cryptochrome (tryptophan tetrad). The red circles indicate the nearest residues, within 5.5 Å, of the flavin cofactor. Additional similarity measures broadly confirming the results from the KLD can be found in the SI, Fig. S2.

The time development of the RMSD calculated for the protein backbone is depicted in Fig. 2A, commencing with the formation of the charge transfer generating the FAD^•–^/Trp_C_^•+^ radical pair. The RMSD is characterized by an effective time constant of 3.0 µs. Over longer time periods, the system plateaus at an RMSD of 3.35 Å, indicative of a stable CRY protein structure and in agreement with previous studies, including one employing the cg-MD paradigm [63], [64]. The flattening out over the course of the simulation indicates the attainment of a stable radical pair structure and no significant protein unfolding, allowing for further analysis of the characteristic conformational changes in terms of an activation response.

We assessed the similarity of each coarse-grained simulation to the dark state simulation, as reported in [64], based on the symmetrized Kullback-Leibler divergence (KLD) between the distributions of positions of each amino acid residue. Fig. 2B shows the average KLD of the radical-pair trajectories as a function of the residue index. A higher divergence value indicates a greater deviation for the respective amino acid residues in the radical pair trajectory relative to the reference Dark State (DS) trajectory. The plot shows marked rearrangements across the entire protein. We will focus on six regions of interest within the ClCry4 structure, chosen based on their large KLD or following previous studies. These regions include the phosphate binding loop (PBL), the C-terminal tail (CT), the EEE region (EEE), and two versatile regions, labelled OPP and CL. The REF region has been included here as a highly conserved region; it comprises an α-helix lining the FAD-cavity. The regions of particular interest are highlighted in Fig. 1. The colour scheme used to highlight regions is consistently applied throughout this work.

One notable region exhibiting significant divergence is the OPP region which has been mentioned in earlier studies [63], [64], though its role and possible functionality has remained elusive. With FAD as the centre, this region (OPP) is located on the opposite side of the protein from the surface tryptophans involved in the electron transfer cascade. The connecting loop (CL) spanning residues 170–200 is another significant region. Situated close to the PBL region, it comprises the connection region of two well-defined α-helices and remains largely disordered, yet appearing important for oligomerization [52]. A third region displaying considerable KLD, highlighted in blue, consists of three consecutive glutamic acids (EEE), previously identified in DmCry as a linear motif at residues 450–452. Interestingly, in a previous equilibrium study, this region displayed substantial divergence during the subsequent photo-reduction step, FAD^•–^/Trp_D_^•+^, while remaining inactive in other redox states involved in the redox cycle [58]. Additionally, we observe minimal divergence in the C-terminal region (CT), spanning residues 470–497.

Previous studies of the FAD^•–^/Trp_D_^•+^ radical pair in ClCry4, both on the all-atom and coarse-grained scale, have indicated that the most prominent rearrangements relative to the dark state centre on the PBL [63], [64]. In contrast, Fig. 2B demonstrates relatively reduced motion in the PBL region, suggesting that its characteristic rearrangement occurs at later stages, possibly relying on the ionisation of the Trp_D_ residue. The latter region is further addressed later with a specific focus on Trp_C_ in relation to the FAD. The region around FAD is generally very conserved, as highlighted in Fig. 2B by the red circles indicating the nearest residues. Visualizing the mean divergence in Fig. 2B on the protein structure, the divergence appears to increase from the centre surrounding the FAD to the protein’s periphery (see Fig. S3 in SI). Additionally, residues 20–33 and 110–125 show noteworthy peaks in the KLD. However, as the biological functionality of these regions is not known at this time, we defer their discussion to the Supporting Information (see Figs. S2).

**Fig. 3:**
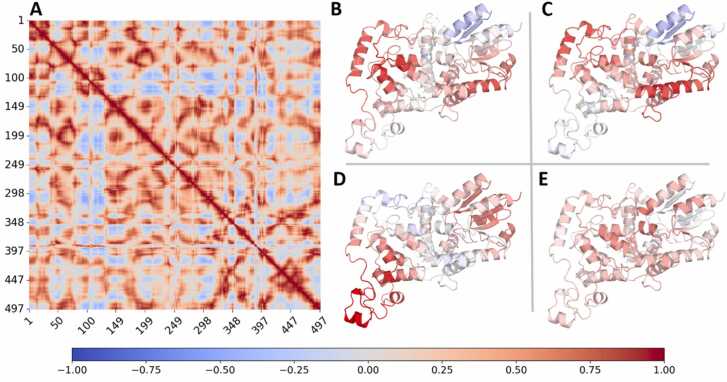
(a) Correlation matrix calculated from the KLD data obtained earlier in Fig. 2 A. (B-E) Average correlation values of protein residues with reference to key regions of interest, i.e. (B) the tryptophan tetrad, (C) the CT region, (D) the EEE region, and (E) the phosphate binding loop (PBL), chosen based on their potential functional roles or structural significance. Strong correlation is observed between the CT region and the tryptophan tetrad. Additional visualization of reference regions on the protein can be found in the SI, Fig. S4.

Fig. 3 summarizes results on the correlation of the observed structural rearrangements over the 20 replica simulations. Fig. 3A displays the correlation matrix of the residue-resolved KLD, i.e. the correlation coefficients of KLDs between various protein residues. The average correlation values across different regions of the protein are shown in Fig. 3B-E, specifically for the tryptophan tetrad (Fig. 3B), the CT region (Fig. 3C), the EEE region (Fig. 3D), and the phosphate-binding loop (Fig. 3E). A close inspection of Fig. 3B reveals that the motion of the tryptophan tetrad exhibits a strongly positive correlation with several branches, most noticeably with the CT region (Fig. 3C). The CT region appears to further be strongly correlated to a surface-exposed α-helix at the opposite side of the protein. In contrast, the PBL displays a weak correlation with both the central FAD region and other protein regions, while Fig. 3D shows a strong localized correlation for the EEE.

**Fig. 4:**
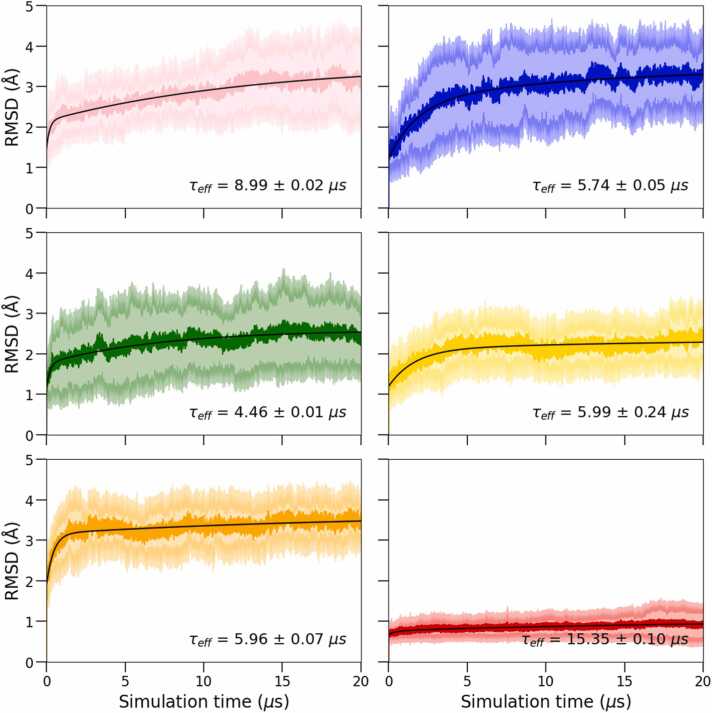
The average RMSD and standard deviation of the regions of interest over the 20 replica simulations are displayed as a function of time after the introduced charge separation in the protein. The colours correspond to the highlighted regions of the protein structure in Fig. 1. A bi-exponential fit (black lines) was chosen to capture the complex time-dependent behaviour of RMSD changes. The bold colours represent the mean RMSD, while transparent colours depict the standard deviation. The fitting function and parameters are summarized in Table S1 of the SI. The effective time constants are displayed. Additional regions are analysed in Fig. S5. Overall, the figure illustrates the temporal evolution of structural fluctuations within the protein, with initial changes stabilizing over time.

Building on the observations from Fig. 3, we further examined the time-dependence of the structural rearrangements associated with the selected regions in the ClCry4 protein. Fig. 4 shows the RMSD calculated for the individual regions as a function of simulation time, along with a biexponential fit. Parameters of biexponential fits of the time-dependence of the RMSD and effective time constants are summarized in Table S1, available in the SI.

The following picture emerges from the analysis of the effective transition times, i.e. the weighted mean of time constants from the biexponential fits. The EEE region (highlighted in blue, Fig. 4) exhibits an apparent increase in the RMSD within the first 4 µs, which proceeds to flatten out over the remainder of the time course. In contrast, the PBL region (pink) demonstrates a much slower overall response, characterized by a gradual increase in RMSD throughout the simulation time and a noticeable rise in the standard deviation after 12 µs. Thus, the PBL appears as a slower responder, only gradually assuming the prominent flexibility characteristic in equilibrium simulations of FAD^•–^/Trp_D_^•+^. Even if less pronounced compared to the previous studies, the FAD^•–^/Trp_C_^•+^ charge shift studied here appears sufficient to induce some structural transition in the PBL on the microsecond timescale. Interestingly, the CT (green) has a much lower RMSD than one might have expected based on its ascribed central role in CRY signaling [50], [57], [60], [62]. Apart from a fast initial transient, the RMSD of the CT region grows slowly over the accessible 20 µs with considerable standard deviation over the entire time course but remains below the RMSD values observed for the PBL, EEE and CL regions. Finally, both the OPP and CL regions (yellow and orange in Fig. 4, respectively) show a quick transient followed by a quasi-steady state with minimal deviations from the average value. As expected, the REF-region (highlighted red in Fig. 4), featuring the α-helix adjacent to the FAD, shows very small RMSD pertaining to the stability around this region. Additional regions of interest, i.e. residues 20–33 and 110–125, are also analysed and can be found in Fig. S5 along with their corresponding effective time constants.

**Fig. 5:**
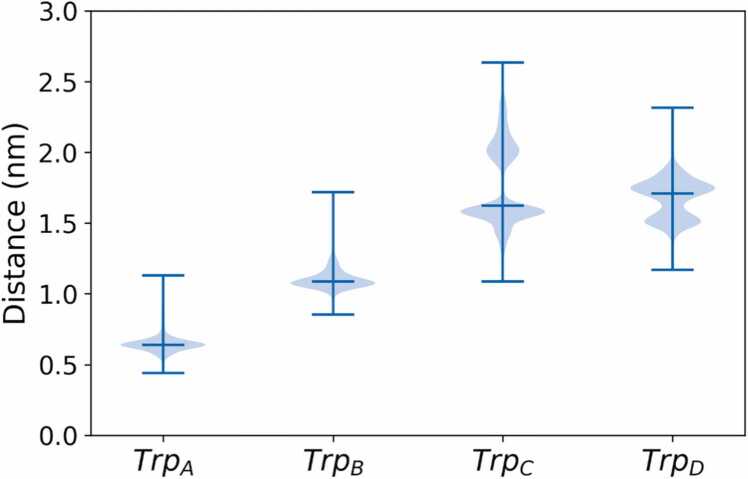
Distributions of the distances between the centres of mass of the isoalloxazine ring of flavin cofactor (FLA1–5 beads) and the four tryptophans involved in the electron transfer cascade (Trp_A_, Trp_B_, Trp_C_, Trp_D_). For each tryptophan, the plot shows the distribution of distances sampled from the collection of 20 trajectories of 20 µs each. The lines extend to the minimum and maximum distances and the mean interposed between them. The individual distributions for single trajectories can be found in the SI, Fig. S6, along with indications of the associated initial positions. The distances from FAD to Trp_C_ and Trp_D_ show a bimodal distribution reflecting a rearrangement in the side chains of these tryptophan residues.

We shift our focus to the distances between FAD and the tryptophan residues involved in the electron transfer cascade, which are central to cryptochromes’ charge separation and putative magnetic field reception. Fig. 5 displays the distributions of distances between the FAD and each consecutive tryptophan, specifically between the centre of mass of the isoalloxazine ring of the flavin cofactor (FLA1–5 beads) and the centre of mass for each tryptophan involved in the electron transfer process. Similar to previous studies [57], [63], [64], the distances between the isoalloxazine ring of the flavin cofactor and each tryptophan increased with the position along the tryptophan tetrad. However, as already noted earlier [64], Trp_C_ and Trp_D_ can switch relative positions, which here occurred in 60 % of all trajectories over the time course of the simulation. Additionally, all trajectories that exhibited rearrangement of Trp_C_ and Trp_D_, accompanied by an increase in the distance of Trp_C_, showed no reversion to the initial starting distance over the total simulation time. As a consequence, the distance distributions of Trp_C_ and Trp_D_ are bimodal. Further analysis of individual distributions in Fig. S6, showed no clear correlation between the starting point of the trajectories and the tryptophan flipping event.

**Fig. 6:**
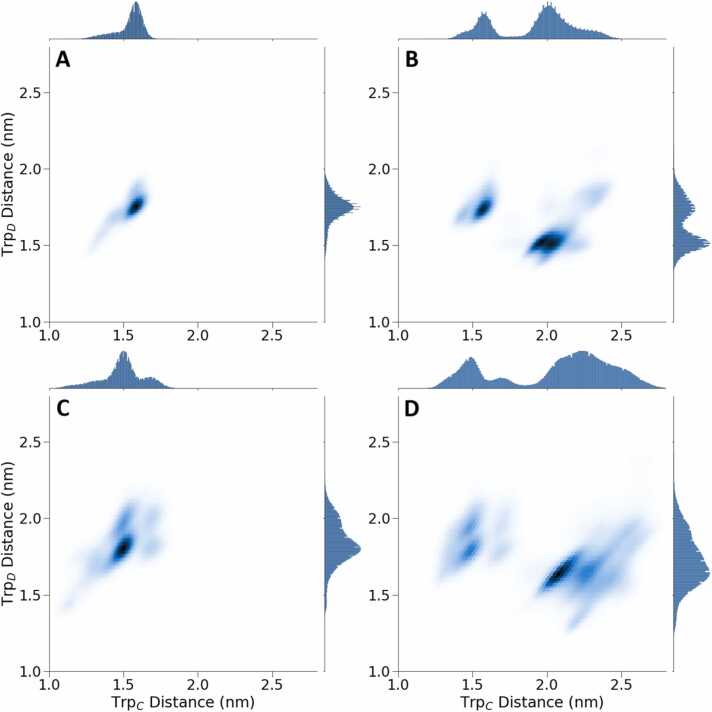
Density plots illustrating the joint probability distribution of Trp_C_ and Trp_D_ distances involved in the electron transfer cascade to FAD. The plots use 2D histograms with hexagonal bins to represent the distribution of distances. Panels A and B display the distances between the centre of mass of FAD beads (FLA1–5) and tryptophan residues, while panels C and D display the distances between FLA2 bead and SC2 bead on each tryptophan residue. The left-sided panels (A, C) focus exclusively on trajectories with no jump in Trp_C_ distance, indicative of the Trp_C_/Trp_D_-rearrangement, while the right-sided panels (B, D) consider trajectories where a reorganisation occurs.

To assist the further inspection of the bimodal distribution of the Trp_C_ and Trp_D_-distances to the flavin observed above, Fig. 6 presents 2D density plots based on these distances, offering a more comprehensive view of the distribution patterns. Figs. 6A and 6B display the centre-of-mass distances of the isoalloxazine ring of FAD and the entire tryptophan, while Figs. 6C and 6D focus on the distances between the FLA2 bead of the FAD from SC2 bead of the tryptophan, which more closely correspond to the centres of spin density of the radical pair. The results in Figs. 6A and 6C are derived from trajectories where Trp_C_/Trp_D_ remain at their initial configuration throughout the simulations, while the results in in Figs. 6B and 6D represent trajectories in which the positions of Trp_C_/Trp_D_ rearrange during the simulations.

We can see that the bimodal distributions occur in Figs. 6B and 6D due to an increase in the Trp_C_ distance with a corresponding decrease in Trp_D_ distance measured from the FAD, i.e. the residues formally switch their order in the chain. It is particularly clear when separately analysing the distributions for the simulations where the Trp_C_/Trp_D_ rearrangement is either present or absent. To determine the rate of this transition, we analysed the lifetime of the distribution of the original form. This analysis, which is detailed in the SI, Fig. S7, reveals a characteristic time constant (τ) of 7.5 µs for the Trp_C_/Trp_D_ rearrangement. Upon detailed inspection of selected structural motifs of the ClCry4 structure, this Trp_C_/Trp_D_ rearrangement is understood to result from a change in the sidechain dihedral angles of the tryptophans, as demonstrated in the SI, Figs. S8-S17. Fig. 6C offers a more detailed view of the structural distribution of the individual tryptophan side chains by displaying pair-wise distances between specific beads characterizing FLA2 and SC2, which closely reflect the centres of spin density of the flavin and tryptophan residues. The four-mode formation observed for small Trp-distances can be attributed to a bimodal distribution of dihedral angles of both tryptophans side chains, Trp_C_ and Trp_D_. In this arrangement, it is energetically more favourable for both tryptophans to position their SC2 bead closest to the FAD.

**Fig. 7:**
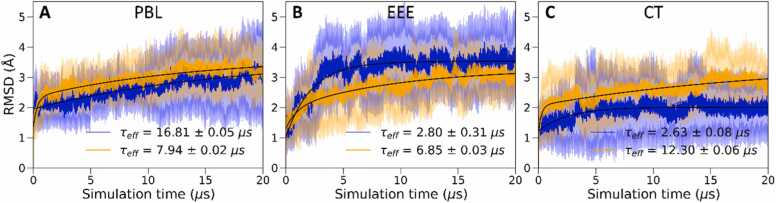
The average RMSD and standard deviation of selected regions of the ClCry4 protein comparing the systems with (orange) and without (blue) the Trp_C_/Trp_D_ rearrangement. (A) Phosphate Binding Loop (PBL), (B) residues 440–460 (EEE) and (C) C-terminal tail (CT). The bold colours represent the mean RMSD, while transparent colours depict the standard deviation. Twelve of the trajectories exhibited the Trp_C_/Trp_D_ rearrangement, and the remaining eight did not. Details of fitting function and parameters can be found in Tab. S2 and S3 of the SI. RMSD values for other regions are provided in the SI, Fig. S7.

We explored whether the rearrangement of the tryptophans Trp_C_ and Trp_D_, as described above, could be correlated to larger-scale structural transformations, both in terms of preceding them or occurring as a result of such changes. To gain some hints about such dynamic correlations and consequences, we studied the RMSD of the pivotal regions, namely the PBL, EEE, and CT regions, comparing the systems with and without the Trp_C_/Trp_D_ rearrangement, with results summarized in Fig. 7 and, for additional protein regions, in the SI, Fig. S18.

Fig. 7A focuses on the PBL region, highlighting the Trp_C_/Trp_D_-rearranging trajectories in orange. This rearrangement manifests a faster effective time constant (τ_eff_), indicating a swifter response time coupled with a slightly higher RMSD. This acceleration in response, particularly evident in the initial microseconds, implies an early-stage reactivity absent in the trajectories lacking this rearrangement. Transitioning to the EEE region, the contrast between the trajectories with and without the Trp_C_/Trp_D_ switch becomes more noticeable. The absence of the rearrangement (Fig. 7B, depicted in blue) displays a more rapid response time, reaching its peak approximately 5 µs before stabilizing. Conversely, the Trp_C_/Trp_D_-rearranging trajectories demonstrate a more gradual increase in RMSD, with a convergence apparent at longer timescales. The CT region presents another intriguing observation. The Trp_C_/Trp_D_ rearrangement consistently maintains a higher RMSD throughout the simulation duration, displaying a steady increase over time, while the absence of the rearrangement remains relatively steady at a lower RMSD. Additionally, two trajectory similarity measures (see in the SI, Fig. S19) showed comparable divergence across residues, with both indicating greater mobility in the C-terminal region. Overall, the observed differences in RMSD values and response times between trajectories with and without the Trp_C_/Trp_D_ rearrangement indicate that even slight alterations in tryptophan side chain configurations may correlate with structural changes within the protein.

## 4 Discussion

Magnetoreception through radical pairs relies on spin dynamics that affect the ratio, i.e. the relative quantum yields of distinct reaction products with discernible downstream effects. In cryptochrome, these differentiating pathways are thought to be provided by the back-reaction, i.e. spin-selective recombination of the radical pair, which leaves the system unaltered, and an absence of such a recombination [13]. The latter is accompanied by stabilization of the radicals, e.g. through proton transfer reactions [22] and structural rearrangement [50], [60], [62], which follow from the altered charge distribution and interactions within the protein. Ultimately, these mid-timescale (faster than what is accessible by all-atom MD but still slow on the timescale of the signal transduction) responses of the activated cryptochrome must innervate a signaling cascade that gives way to biochemical signaling [13]. This process could involve structural responses, such as larger-scale conformational rearrangements and dimerization [81], [82], association with or dissociation from interaction partners [28], or phosphorylation [60], [63], [64]. Additionally, it may entail indirect changes, such as an altered cellular redox status due to the dark part of the photocycle or irreversible degradation [83].

In this overall process, the formation of the primary radical pair through photo-induced electron transfer plays a pivotal point, not only as it initiates magnetosensation via the coherent spin dynamics but also as it gives way to a large-scale charge rearrangement that perturbs the dark-adapted protein structure and initiates a conformational response. With an accessible timescale of tens or hundreds of microseconds, course-grained MD can afford insights into these processes on a timescale that equates to or exceeds that of the coherent spin dynamics, namely microseconds. Although the final processes and structural rearrangements leading to cellular signaling occur on a slower timescale, these events are likely initiated by the earlier, faster responses. Understanding the structural dynamics on the timescale of tens of microseconds can thus provide valuable insights into the initial processes by enabling the identification of key sites.

Limited structural experiments on cryptochrome, such as proteolysis [41], [54] and hydrogen-deuterium exchange mass spectrometry (HDX-MS) [84] studies, indicate light-induced structural adaptations that suggest increased protection in the photo-reduced state. While these findings provide critical insights into cryptochrome dynamics, they capture timescales much longer than those observed in our study.

While the magnetic field does not directly affect the structural dynamics of the Trp_C_, it modulates the lifetime of the radical pair, as the spin dynamics influence whether the pair recombines to the initial dark state, or persists in its radical form. The population we describe comprises radicals that have not yet recombined, allowing us to trace the early stages of spin dynamics and the initial phases of signaling before recombination occurs. As the magnetosensitive spin dynamics occur on a timescale of ∼1 µs, we are thus observing events during and, provided that no recombination has occurred, directly following this window of spin modulation. Although the molecular dynamics themselves are not impacted by the magnetic field, they do influence the spin dynamics, as they induce spin relaxation and influence recombination rate constants [75] by, for instance, modulating the distance between the radical pair partners and the orientation of the radicals relative to the applied magnetic field.

As described above, the protein responds to the charge separation through photo-activation, forming FAD^•–^/Trp_C_^•+^ with structural rearrangements affecting the entire protein and abating with an effective time constant of 3 µs. Key regions of rearrangement are the CL, the EEE region and the CT. The dynamics of the PBL is noteworthy. While the PBL is the single outstanding dynamic feature in equilibrium simulations of the subsequent FAD^•–^/Trp_D_^•+^ state, here it assumes a comparably secondary role, both in terms of a relatively larger resemblance of the dynamics to the dark equilibrium state (as assessed via the KLD) and relatively slow overall response time (it does show a quick response at first, which reflects its considerable intrinsic flexibility, as seen in equilibrium simulations). Overall, these observations suggest that the PBL is not a primary responder to the charge separation in the FAD^•–^/Trp_C_^•+^ and might gain its characteristic flexibility, i.e. the gate opening that grants access to the FAD binding pocket as described earlier [64], only after other structural rearrangements and further charge migration to Trp_D_^•+^. Based on the results of our simulations, one could tentatively suggest that the CL region might activate the PBL, increasing its flexibility to open. The EEE region, which harbours a PDZ-binding motif, exhibits a marked response to the charge separation, displaying a large RMSD relative to the initial state and a considerable KLD relative to the dark state trajectory with a time constant of 5.7 µs. Interestingly, an increase in the standard deviation of the RMSD of the PBL after 12 µs appears to correlate with a decrease in that of the EEE-region, hinting at a correlation and the predecessor role of the EEE-region. However, more detailed studies will be necessary to substantiate the possible connection between the behaviour of the PBL and EEE region. The CT also exhibits a slow response to the charge separation, and its RMSD remains comparably low, suggesting that on a timescale of 20 µs, we only obtain a first glimpse of the large-scale rearrangements eventually seen in experiments. As described in the supporting information (SI), we employed two additional similarity measures, which yielded comparable results (Fig. S2). Notably, there was an increase in divergence in the EEE region and the C-terminal region when compared to the default KLD. The distance distribution of the sidechains of Trp_C_ and Trp_D_ are bimodal due to a simultaneous rearrangement of the side chain dihedral angles, which leads to the tryptophans changing their relative positions within the charge transfer chain. As a result, Trp_C_ shifts away from the FAD, giving rise to a more solvent-exposed position and larger distance fluctuations relative to its more buried initial state. The spread of distances, visualized in Fig. 6, resembles a blurring effect, potentially indicating the entire protein’s large-scale protein motion or breathing motions. Additionally, this Trp_C_/Trp_D_ rearrangement also appears to make Trp_C_ more mobile, aligning with its increased surface exposure. This rearrangement appears to be irreversible on the timescale of the simulations and has affected more than half of the simulation trajectories. Interestingly, in trajectories that undergo the Trp_C_/Trp_D_ rearrangement, the CT exhibits increased mobility, marked by a characteristic rise in RMSD over 12 µs. In contrast, trajectories that maintain their initial Trp positions quickly stabilize, levelling off at a lower RMSD value. This suggests the possibility of another process involved, potentially causing the CT region to be released or inducing additional motion over longer timescales which we only observe at the early stages. The close association between Trp_C_ and Trp_D_ sidechains within the protein could explain this interaction. When the tryptophans undergo the described rearrangement, they untwist, allowing the CT region more freedom to move. Note that we do not want to imply that the untwisting is a prerequisite for the CT release; instead, the untwisting could also result from the CT region's movement. On the other hand, the PBL appears to be uncorrelated to the Trp_C_/Trp_D_-rearrangement, while the EEE-rearrangement proceeds more rapidly when Trp_C_ and Trp_D_ remain in their initial positions. While speculative, the observed interactions between the EEE, Trp_C_/Trp_D_-rearrangement, and CT could reflect an overall order of processes, whereby EEE initiates, profiting from the intact Trp_C_/Trp_D_-arrangement, and the CT follows the Trp_C_/Trp_D_ untwist. For example, and again purely speculative, one could imagine a process where the EEE rearrangements lead to the dissociation of cryptochrome from a PDZ-binding partner at a membrane interface, followed by the release of the CT to recruit signaling partners. In light of recent findings that the C-terminal region of *Drosophila* cryptochrome alone mediates magnetic field responses, it is intriguing to consider whether a similar independent mechanism exists in the C-terminal region of avian cryptochrome 4 or if it requires the activity of other regions, such as the phosphate-binding loop (PBL). However, determining whether the tail alone functions as a transducer in birds remains challenging without understanding the specific interaction partners involved, which should be addressed through further experimental investigation.

While it will be interesting to follow up on these ideas, it will be more pertinent to assess the fidelity and flipping propensity in all-atom models, whereby, given the involved long timescales, free energy perturbation methods will likely be necessary. Note that in an earlier study [64], this rearrangement was also present in the dark state, suggesting it may be an inherent characteristic of these proteins rather than exclusively associated with the formation of the radical pair. Future studies will quantify the relative ease of the untwisting in various redox states and thus relevance clarify its relevance as part of a dedicated activation processes.

**Fig. 8:**
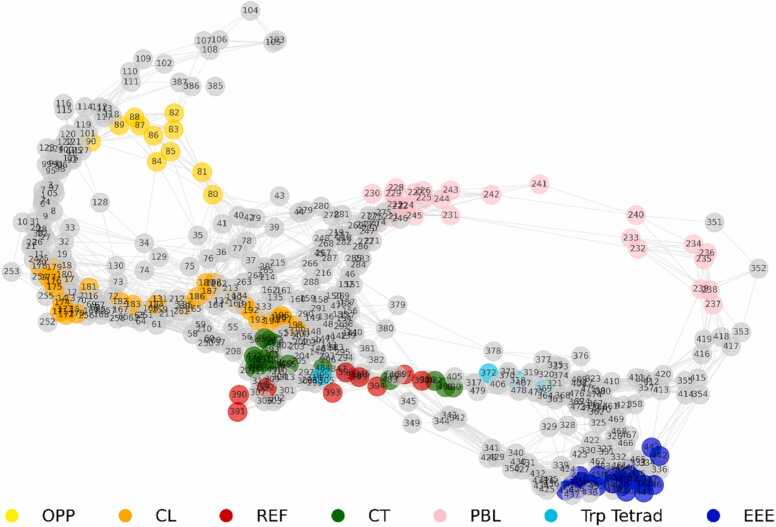
Graph resulting from the correlation matrix of the normalized KLD for all trajectories. The threshold of 0.86 applied to filter the correlation matrix, highlighting only the strong correlations among the trajectories. The colours correspond to the same regions of interest as in previous figures: OPP: 80–90 (yellow), CL: 170–200 (orange), PBL: 220–245 (pink), REF: 388–399 (red), EEE: 440–460 (blue) and CT: 480–497 (green), tryptophan tetrad: W395, W372, W318, W369 (cyan). Please refer to Figs. S20-22 for additional insights in terms of communities and centralities.

Lastly, we aim for a more holistic view of the protein activation dynamics. To this end, we expanded upon the similarity data analysed in Fig. 3A by processing the correlation matrix from all twenty simulated trajectories. As mentioned above, each trajectory was aligned to the DS, and the KLD similarity measures were computed relative to this state. Employing a fixed filtering threshold, the correlation matrix was translated into an adjacency matrix informing a network topology; amino acid residues are hence considered nodes in the graph, which are connected by an edge if their respective KLDs are correlated. Fig. 8 shows the resulting graph.

To facilitate the identification of the various protein regions, each was highlighted according to the used colour scheme, see Fig. 1, with the four remote tryptophans involved in the electron cascade, Trp_A_, Trp_B_, Trp_C_, and Trp_D_, additionally highlighted in cyan. Examination reveals a network of connections between these tryptophans within the protein: an immediate pathway involving the CT (green) and REF (red) region facilitated by intermediate residues, two distant pathways connecting to the PBL and EEE region and a remote pathway extending to other protein regions, including OPP and CL. The insights regarding the pathways involving CT, EEE and PBL regions, observed from Fig. 8, are supported by the characteristic time constants (τ) calculated for each protein region depicted in Fig. 4 and detailed in the SI, Table S1. As expected, based on the network analysis, the CT region exhibits a rapid response, with a τ-value of (4.45 ± 0.01) µs with EEE region following behind (5.7 ± 0.05) µs. At the same time, the PBL displays the slowest response, with a τ value of (9.0 ± 0.02) µs. We also note that the characteristic time constants mentioned earlier suggest a balance, where the order of the timescale for the radical-pair mixing to generate efficient magnetic-field-effect sensing is on the order of 1 µs (electron spin precession time in the geomagnetic field is on the order of 720 ns [13]) and the system, specifically the phosphate-binding loop, takes around 10 µs to activate. This timing ensures the initial magnetic sensing is followed by a prompt yet sufficiently delayed activation step, allowing for effective signal transduction. In turn, the delayed activation facilitates solvent access to the protein centre, likely resetting the system to its initial ground state by enabling re-oxidation of flavin.

Though we have provided characteristics times of processes following the photo-activation on the timescale of 10 µs, the real cellular environment is complicated and structural responses are expected to emerge over longer times and in dependence of the environment, including interaction partners [27], [28], [85], temperature and pH. As for the latter, we have chosen a protonation state that reflect the pH in cells, initiated the simulations from the crystal structure, and assumed a temperature of 310 K, in line with the bird's temperature. Though we cannot model a system of realistic complexity at this current stage, the core protein structure is not expected to be affected by interactions and minor changes in the cellular environment, and thus the results obtained on this simplified system are still representative.

## 5 Conclusion

In this study, we employed a coarse-grained MD approach to investigate the early-stage dynamics of bird cryptochrome 4 photoreduction. By analysing 20 replications of 20 μs each, we traced the non-equilibrium processes following the formation of the FAD^•–^/Trp_C_^•+^ radical pair, implicated in light-induced magneto-sensing. Our findings reveal that the charge separation induces significant structural rearrangements across the protein, characterized by an overall time constant of 3 µs. However, several key protein regions exhibited slower responses with larger characteristic time constants.

We found that the C-terminal region (CT) displayed the fastest specific response, with time constant of 4.5 μs, while the EEE-region, containing a PDZ-binding motif, followed closely behind with 5.7 μs. These results suggest that these regions could potentially influence or precede the dynamic behaviour of other areas, such as the phosphate-binding loop (PBL). Notably, the PBL exhibited a smaller dynamic flexibility than expected based on its equilibrium behaviour in the FAD^•–^/Trp_D_^•+^ redox state and a slower response time of 9 μs.

We utilize a unique network-analysis approach to discern the dynamics of the activation processes. To this end, correlation in the divergence of the individual replica trajectories from the dark-state dynamics, as assessed by a distance measure based on the Kullback-Leibler divergence, serves to inform a graph representation of the activation. This network analysis of correlated residue motion relative to the dark state revealed a direct pathway connecting the tryptophan tetrad to the CT, as well as two distant pathways connecting the EEE and PBL regions, further supporting their likely interactions. This suggests that the PBL is a slower or secondary responder, likely aligning with the hypothesis that the opening of the “gate” to the FAD facilitated by this region is relevant to the regeneration of the photo-reduced FAD, such as by permitting access to oxidants. The relatively slow timescale on the order of 10 µs appears well-balanced relative to the 1 µs timescale of the magnetosensitive spin dynamics, suggesting that spin dynamics precede the protein's structural dynamics leading to sensor regeneration.

The study also revealed a rearrangement of two tryptophan residues, Trp_C_ and Trp_D_, involved in the formation of the correlated radical pair. Their side chains switch relative positions while preserving the necessary distances for electron transfer processes. This Trp_C_/Trp_D_ rearrangement led to subtle effects on various protein regions, with the most significant impact observed in the CT. In trajectories where this rearrangement is apparent, the CT exhibits both a higher RMSD and a steady increase in RMSD over time, indicating distinct dynamic characteristics compared to the initial arrangement.

Overall, this study has highlighted regions of interest within the ClCry4 protein and the subtle motions occurring due to the tryptophan residues involved in the electron cascade. These findings provide insights into the order of events across the protein and suggest potential targets for further investigation into the mechanisms underlying light-induced magnetosensation in birds.

## Data Access Statement

Data underpinning this publication are available from the Supplementary material. Molecular dynamics trajectories will be provided upon reasonable request.

## CRediT authorship contribution statement

**Daniel Kattnig:** Writing – review & editing, Supervision, Project administration, Investigation, Funding acquisition, Conceptualization. **Ilia A. Solov’yov:** Writing – review & editing, Supervision, Project administration, Funding acquisition. **Fabian Schuhmann:** Writing – review & editing, Investigation, Formal analysis, Data curation. **Jessica L. Ramsay:** Writing – original draft, Visualization, Investigation, Formal analysis, Data curation.

## Declaration of Competing Interest

The authors declare that they have no known competing financial interests or personal relationships that could have appeared to influence the work reported in this paper.

## Appendix A Supplementary material

.

Analysis codes and cg-MD simulation inputs (including initial molecular structures, parameters and topology files) and outputs (final molecular structures) of the 20 replicas.
