# Supplementary material for: Cryptochrome magnetoreception: Time course of photoactivation from non-equilibrium coarse-grained molecular dynamics
Source: Comput Struct Biotechnol J. 2024 Nov 10;26:58–69. doi: 10.1016/j.csbj.2024.11.001 (PMC11725172; doi:10.1016/j.csbj.2024.11.001)
Supplement: Supplementary file 1 — Supplementary material [file mmc1.pdf]

Supplementary Information

Cryptochrome magnetoreception: time course of photoactivation from non-equilibrium coarse-grained molecular dynamics

Jessica L. Ramsay[1][2], Fabian Schuhmann[3], Ilia A. Solov'yov[4][5][6], Daniel R. Kattnig[1][2],*

[1] Department of Physics, University of Exeter, Stocker Rd., Exeter EX4 4QL, U.K.
[2] Living Systems Institute, University of Exeter, Stocker Rd., Exeter EX4 4QD, U.K.
[3] Niels Bohr International Academy, Niels Bohr Institute, University of Copenhagen, Blegdamsvej 17, Copenhagen 2100, Denmark.
[4] Institute of Physics, Carl von Ossietzky Universität Oldenburg, Carl-von-Ossietzky Str. 9-11, Oldenburg 26129, Germany.
[5] Research Centre for Neurosensory Science, Carl von Ossietzky Universität Oldenburg, Carl-von-Ossietzky-Str. 9-11, Oldenburg 26129, Germany.
[6] Center for Nanoscale Dynamics (CENAD), Carl von Ossietzky Universität Oldenburg, Ammerländer Heerstr. 114-118, Oldenburg 26129, Germany.

[1] RMSD Whole Protein: Individual Trajectories

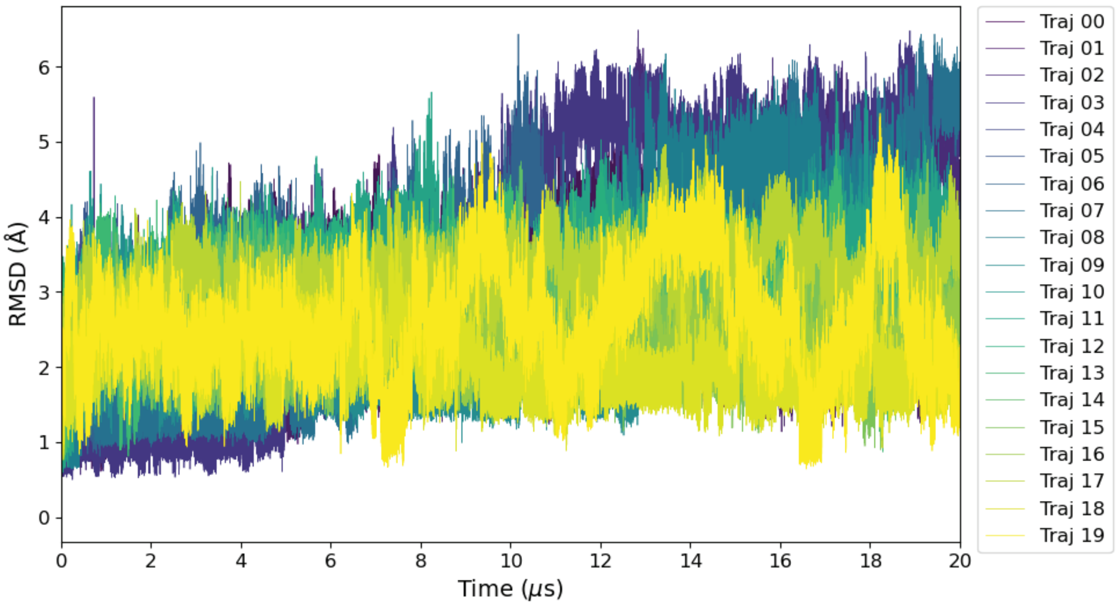

Figure S1: The time evolution of the root-mean-square deviation (RMSD) computed for all simulation trajectories referenced and aligned to the Dark State (DS) simulation (i.e. frame 1). The RMSD for the whole backbone of the protein is shown. The values remain at the same level throughout the simulation indicating a stable structure.

[2] Similarity Measures: Wasserstein Distance, Discrete Fréchet Distance

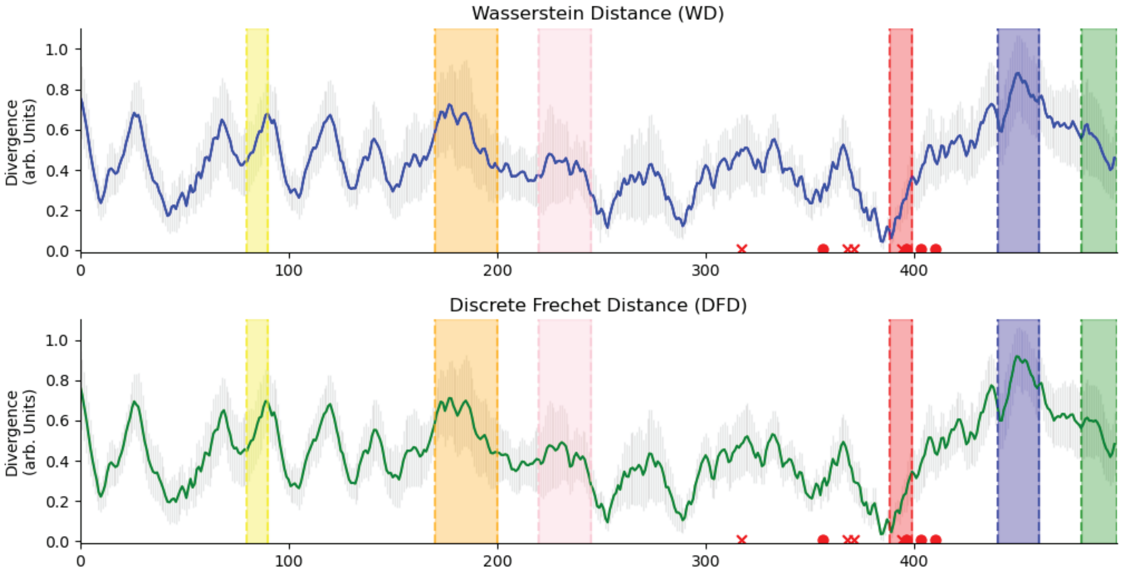

Figure S2: Average Wasserstein Distance (WD; blue) and Discrete Fréchet Distance (DFD; green) of the coarse-grained RPC trajectories relative to the DS equilibrium simulation, with standard deviation shown as grey sticks. Significant motion is observed around residues 440-460 (highlighted in blue, denoted EEE), which contains a linear EEE motif. Other regions displayed include residues 170-200 (highlighted in orange, denoted CL) and 80-90 (highlighted in yellow, denoted OPP), with the latter located roughly opposite the surface exposed tryptophan, Trp_D. Both the phosphate-binding loop (highlighted in pink, denoted PBL) and CT (highlighted in green, denoted CT) display less motion than other regions of the protein.

[3] Similarity Measure: Average KLD visualised on the protein structure

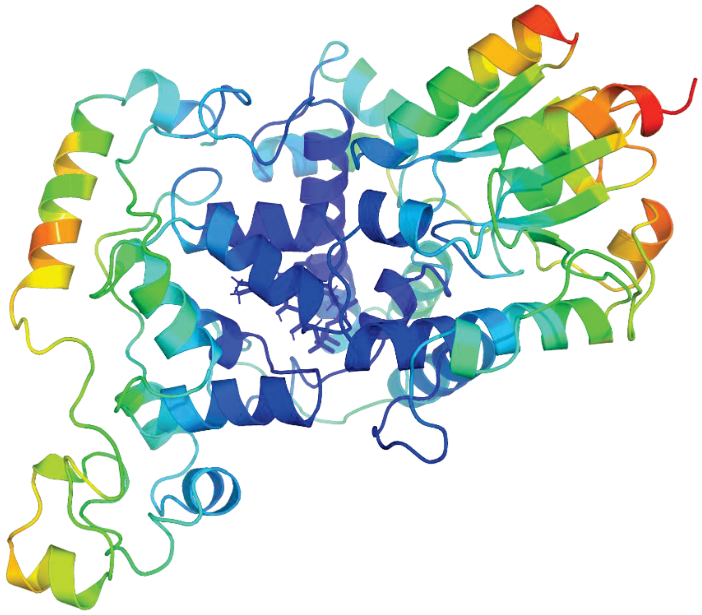

Figure S3: The Average KLD (Kullback-Leibler divergence) of the coarse-grained trajectories, where each simulation is compared to the DS. This is the same data shown in manuscript, Figure 2b, visualised on the protein structure. We see that KLD increases the further away from the protein's centre, with the warmer colours indicated larger divergence. With the scale from 0 to 1 (arb. units) blue indicates values close to zero, whilst red indicates values close to 1.

[4] Average correlation values of protein residues with reference to CL and OPP, respectively

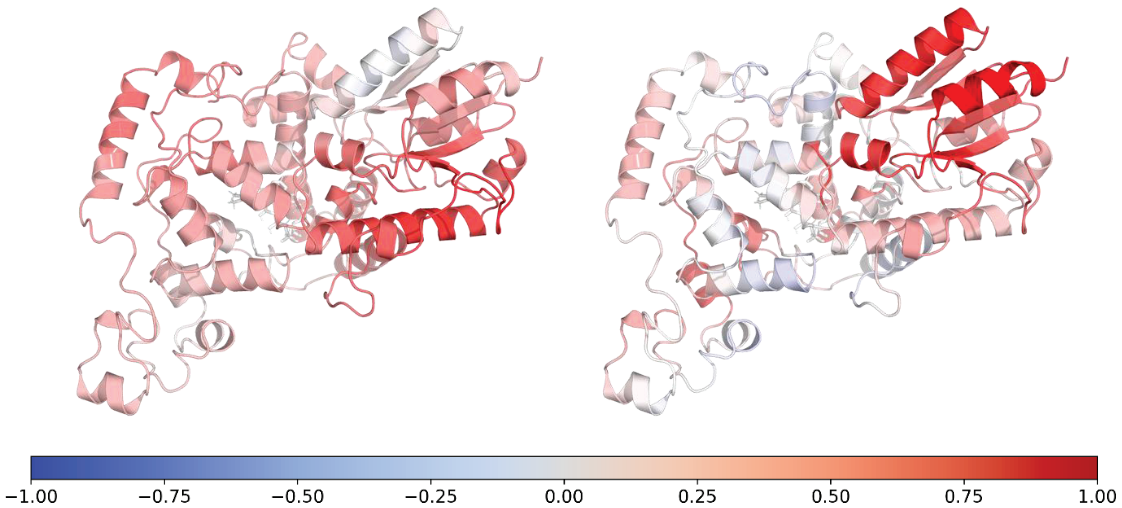

Figure S4: Average correlation values of protein residues with reference to (a) Connecting Loop (CL: 170-200) (b) region opposite surface exposed tryptophan (OPP: residues 80-90). The red, warmer colours corresponding to strong positive correlation.

[5] Table of the parameter values and the fitting of the data

The bi-exponential fit function used for Fig. 4 and Fig. 7:

$$y = A\left(1 - P\exp\left(-\frac{t}{\tau_1}\right) - (1-P)\exp\left(-\frac{t}{\tau_2}\right)\right) + C \tag{1}$$

The tau effective is calculated:

$$\tau_{eff} = P\,\tau_1 + (1-P)\,\tau_2 \tag{2}$$

Table S1: The parameter values for the average RMSD for calculated for each protein region. The underlying data are plotted in the manuscript, Figure 4 and, for additional regions, in Fig. S5.

Region	A	P	C	τ_1 (μs)	τ_2 (μs)	τ_{eff} (μs)
Whole Protein	1.6597± 0.006	0.3571± 0.0003	1.6947± 0.0006	0.3443± 0.0007	4.4663±0.0024	2.9942± 0.0019
PBL	2.0891±0.022	0.3258±0.0006	1.4676± 0.0019	0.1962±0.0009	13.2338± 0.0249	8.9859±0.0187
EEE	2.2248±0.028	0.6139± 0.0009	1.2319± 0.0008	1.8265± 0.0054	11.959± 0.1259	5.7384± 0.0496
CT	1.4275± 0.019	0.417± 0.0008	1.1697± 0.0019	0.2221± 0.0012	7.49±0.0139	4.4594± 0.0098
OPP	1.1929± 0.069	0.7612± 0.0029	1.197± 0.0058	1.654± 0.0058	19.8061±0.9644	5.59891± 0.236
CL	1.7567± 0.033	0.7178± 0.0012	1.901±0.0013	0.4464± 0.0008	20±0.223	5.9647±0.0669
REF	0.3534±0.001	0.2383±0.0009	0.6817±0.0004	0.4891±0.0041	20±0.1297	15.3496±0.1005
A1	1.3069±0.034	0.3514±0.001	1.0969±0.0014	0.4394±0.0023	20±0.1338	13.1272±0.0888
B1	1.6372±0.0007	0.9991±1425.0031	1.3984±0.0006	2.7159±3.4468	2.7159±0.0024	2.7135±4.8708
B2	0.9877±0.0016	1.00±0.0015	1.6288±0.0013	1.2858±0.0035	15.0967±0.000	1.2858±0.0203
D1	0.7789±0.0008	0.4999±0.0011	1.1153±0.0008	0.5253±0.0026	3.9378±0.0106	2.2318±0.0065

Table S2: The parameter values for the average RMSD for only the trajectories where the Trp_C/Trp_D re-arrangement is absent for each protein region. The data are plotted in Fig. 7 in the manuscript, and Fig. S18.

Region	A	P	C	τ_1 (µs)	τ_2 (µs)	τ_{eff} (µs)
Whole Protein	1.5038±0.013	0.3384±0.006	1.6716±0.014	0.1693±0.0008	3.2649±0.0024	2.2174±0.0025
PBL	1.6619±0.025	0.0554±38.4004	1.9509±0.005	16.8131±<0.0001	16.8143±<0.0001	16.8143±0.0484
EEE	2.5447±0.0182	0.9667±0.043	1.0203±0.014	2.2022±0.007	20±8.9851	2.7954± 0.3091
CT	0.78±0.011	1±0.1385	1.2274±0.001	2.6297±0.0597	2.2141±<0.0001	2.6297± 0.0829
OPP	1.3134±0.143	0.9471±0.074	0.9785±0.0016	1.6898±0.0086	19.9986±8.2089	2.659± 0.4552
CL	2.0892±0.049	0.7924±0.016	1.9056±0.0021	0.4013±0.0009	20±0.3679	4.4699± 0.0825
A1	0.9855±0.0059	0.5442±0.0028	1.2245±0.0019	0.6016±0.0042	20±0.4567	9.4426±0.2150
B1	1.9182±0.0013	1±0.0243	1.0107±0.0011	2.5840±0.0181	3.4016±0.000	2.5840±0.0269
B2	1.0914±0.0034	0.7510±0.0028	1.5078±0.0034	0.2597±0.0025	2.0425±0.0238	0.07037±0.0079
D1	0.7296±0.0034	0.6898±0.0032	1.0270±0.0034	1.9512±0.0041	0.0577±0.0014	1.3638±0.0067

Table S3: The parameter values for the average RMSD for only the trajectories where the Trp_C/Trp_D re-arrangement. The data are plotted in Fig. 7 in the manuscript, and Fig. S18.

Region	A	P	C	τ_1 (µs)	τ_2 (µs)	τ_{eff} (µs)
Whole Protein	1.8102±0.0008	0.3783±0.0003	1.6825±0.0008	0.4677±0.0011	5.5089±0.0047	3.6021±0.0034
PBL	2.2872±0.0025	0.4259±0.0005	1.3625±0.0019	0.3495±0.0012	13.5777±0.0414	7.9439±0.0248
EEE	1.9926±0.0019	0.3351±0.0007	1.3021±0.0014	0.9399±0.0052	9.8213± 0.0367	6.8452±0.0253
CT	2.3007±0.0046	0.3899±0.0009	1.1625±0.0024	0.2584±0.0011	20±0.0987	12.303±0.0626
OPP	1.1314±0.0064	0.6973±0.0027	1.2732±0.0009	1.4642±0.0055	20± 0.7309	7.0757±0.2268
CL	1.5377±0.0046	0.6524±0.0017	1.8939±0.0017	0.4995±0.0015	20± 0.2895	7.2782±0.1058
A1	1.5390±0.0034	0.2820±0.0009	0.9896±0.0016	0.3215±0.0020	20±0.0979	14.4511±0.0724
B1	1.9345±0.0026	0.3289±0.001	1.1834±0.0026	0.2143±0.0016	3.2280±0.0041	2.2369±0.004
B2	1.2160±0.0024	1±0.0018	1.4139±0.0019	1.3511±0.0043	16.4901±0.0000	1.3511±0.0278
D1	0.9482±0.0009	0.5611±0.0007	1.0593±0.0009	0.5030±0.0019	5.9294±0.0179	2.8849±0.0087

[6] Additional RMSD Regions

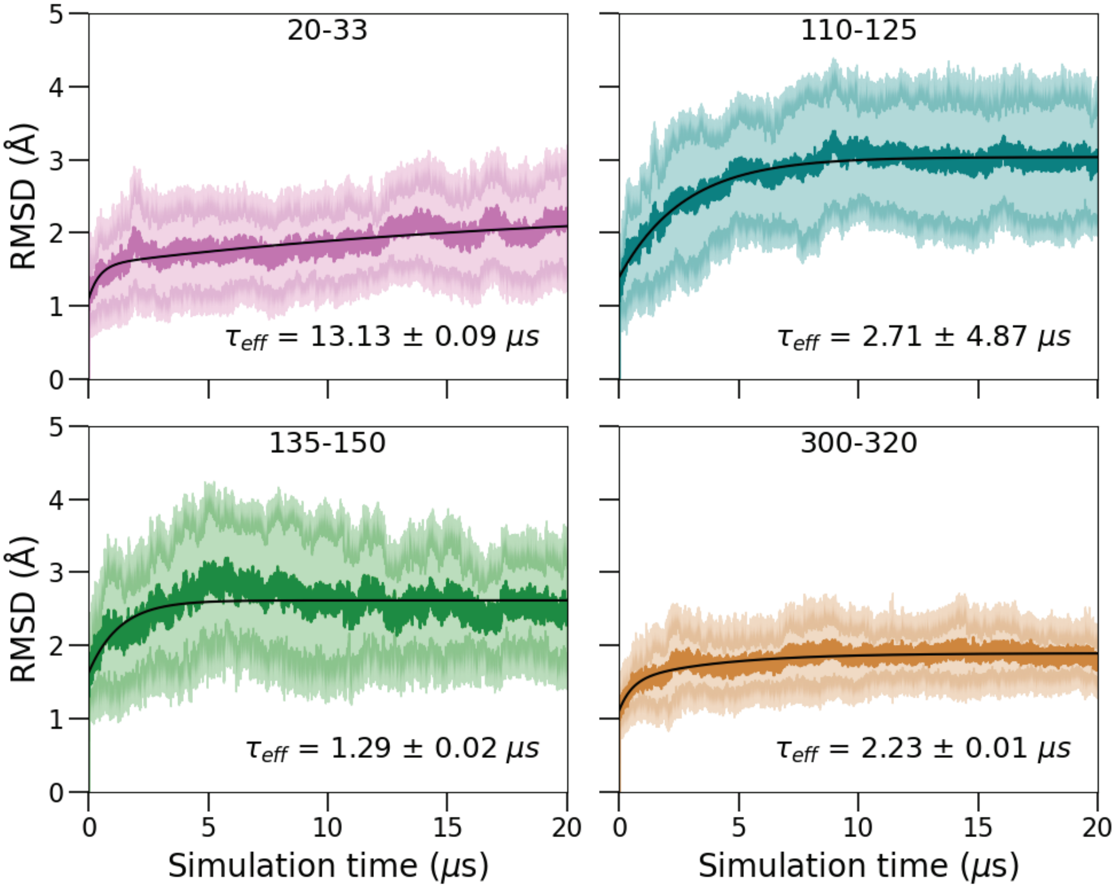

Figure S5: The average RMSD and standard deviation of additional regions of interest are displayed as a function of time after the introduced charge separation in the protein for all twenty trajectories. A bi-exponential fit (black lines) was chosen to capture the complex time-dependent behaviour of RMSD changes. The bold colours represent the mean RMSD, while transparent colours depict the standard deviation. The residue ranges are listed at the top of each subfigure. The effective time constants are displayed.

[7] Individual distributions of each tryptophan

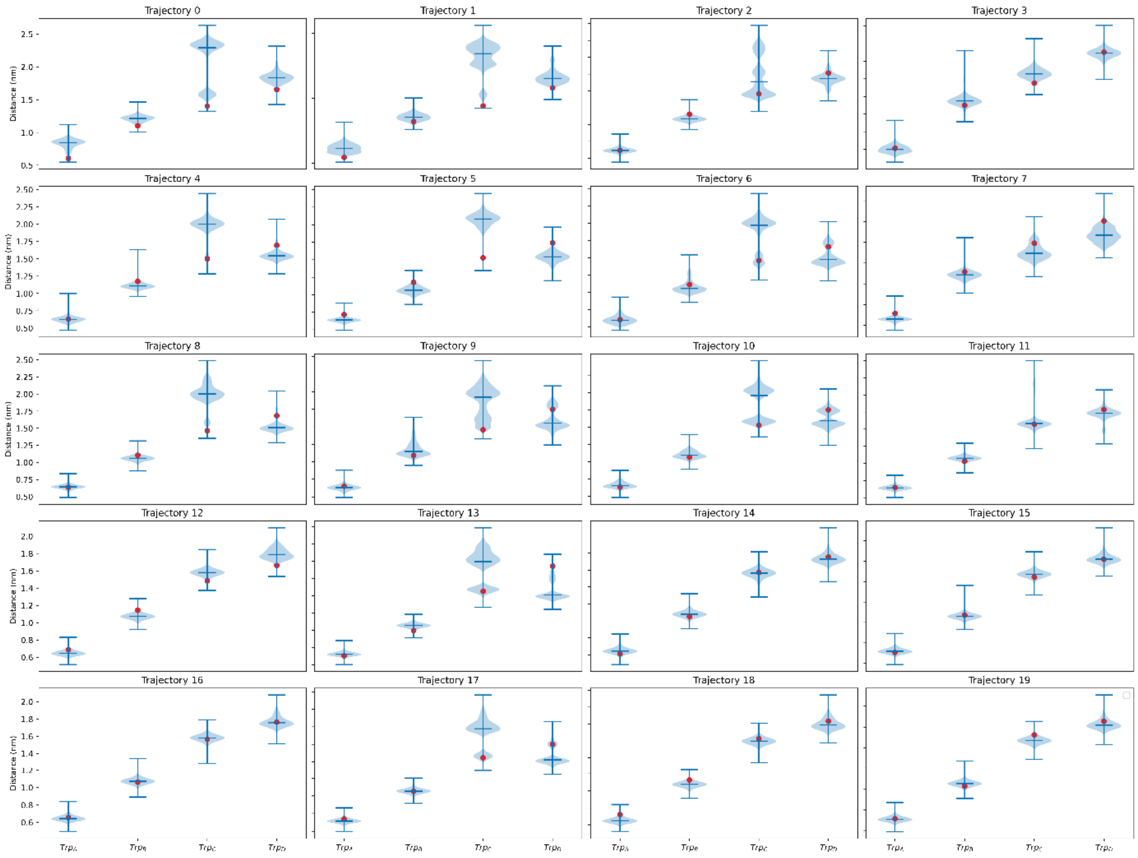

Figure S6: Distributions of the distances calculated between the centres of mass of the isoalloxazine ring of flavin cofactor (FLA1-5 beads) and the four tryptophans involved in the electron transfer chain (Trp_A, Trp_B, Trp_C, Trp_D). The lines extend to the minimum and maximum distance and the mean interposed between them. The red dot indicates the initial position of each tryptophan for each trajectory.

[8] The lifetime of flipped configuration. Add text.

A constraint was applied onto the exponential fit to ensure that the fit goes through 20, the considered number of trajectories, at time 0.

$$y = A \cdot e^{-\frac{t}{\tau}} + C \tag{3}$$

Where $C = 20 - A$.

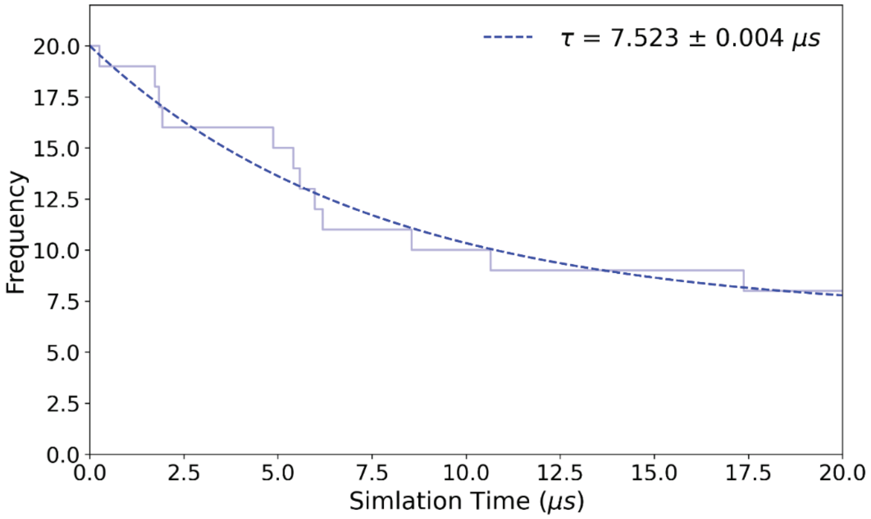

Figure S7: The frequency for Trp_C/Trp_D rearrangement over simulation time during the activation process. The data is fitted to an exponential function. The parameter values include: {A: 13.14, C: 6.86}. The time constant (τ) is displayed on the figure.

[9] Joint distribution plots comparing the Trp_C and Trp_D distance

[a] All 20 trajectories

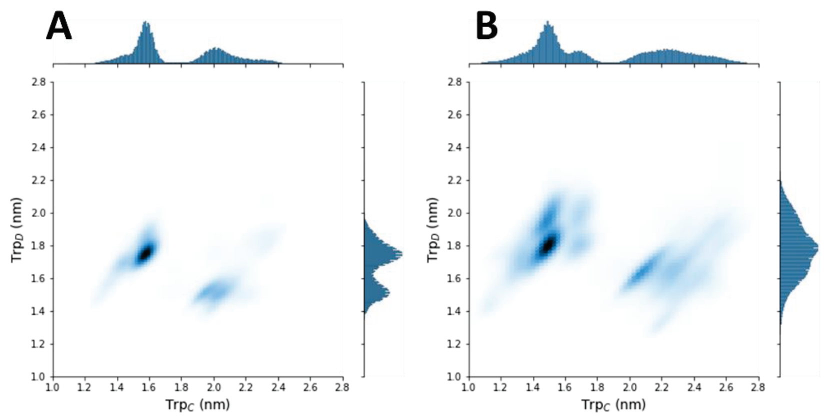

Figure S8: The distances of TrpC and TrpD calculated with respect to FAD using two distinct methods. Panel (a) depicts the centre-of-mass distances between the isoalloxazine ring of FAD (FLA1-5) and the entire tryptophan residue, providing an overall view of their spatial relationship. Panel (b) focuses on the distances between bead FLA2 of FAD and bead SC2 of tryptophan, which more closely represent the centres of spin density of the radical.

[b] Comparing Trp_C and Trp_D distances for the initial and final microsecond

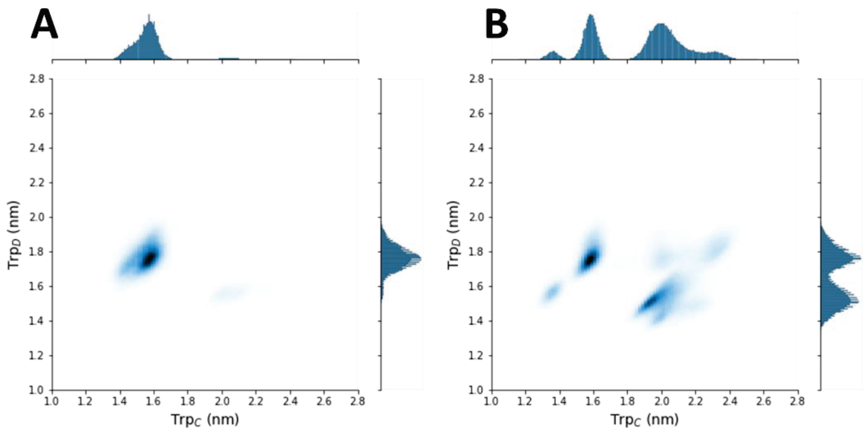

Figure S9: The distance of Trp_C and Trp_D calculated with respect to FAD using the centre-of-mass distances of the isoalloxazine ring of FAD (FLA1-5) and the entire tryptophan. Panel (a) displays the initial microsecond (0-1 μs) of the simulation, while panel (b) represents the final microsecond (19-20 μs).

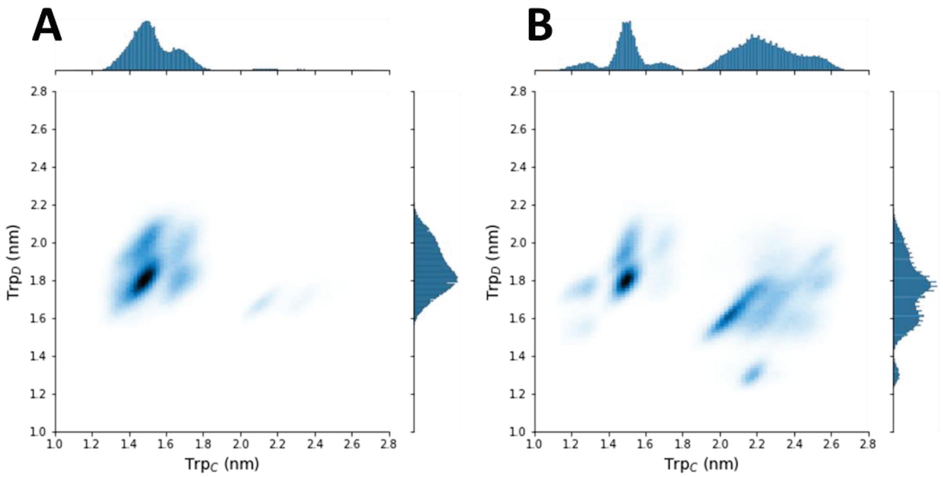

Figure S10: The distance of Trp$_C$ and Trp$_D$ calculated with respect to FAD using the distances of bead FLA2 of FAD from bead SC2 of tryptophan, which more closely correspond to the centres of spin density of the radical. Panel (a) displays the initial microsecond (0-1 µs) of the simulation, while panel (b) represents the final microsecond (19-20 µs).

[10] Comparison of Distance and Dihedral Angles: Joint Distribution

[a] TrpC

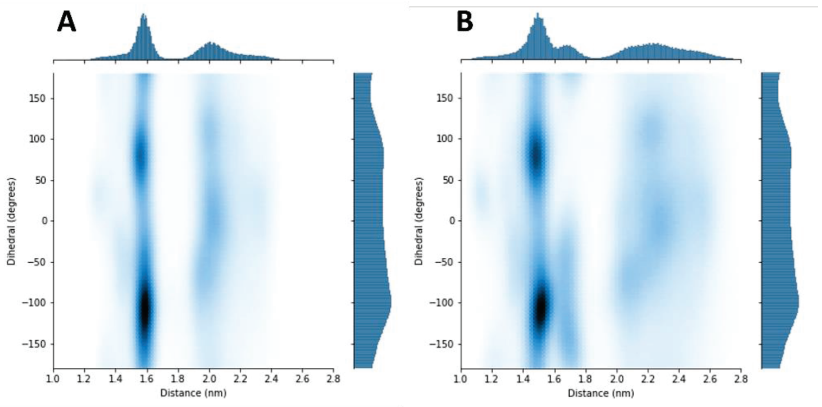

Figure S11: Joint distribution functions comparing the dihedral angle and distance of Trp_C calculated with respect to FAD using two different distance methods. Panel (a) depicts the centre-of-mass distances between the isoalloxazine ring of FAD (FLA1-5) and the entire tryptophan residue. Panel (b) focuses on the distances between bead FLA2 of FAD and bead SC2 of tryptophan, which more closely represent the centres of spin density of the radical.

[b] TrpD

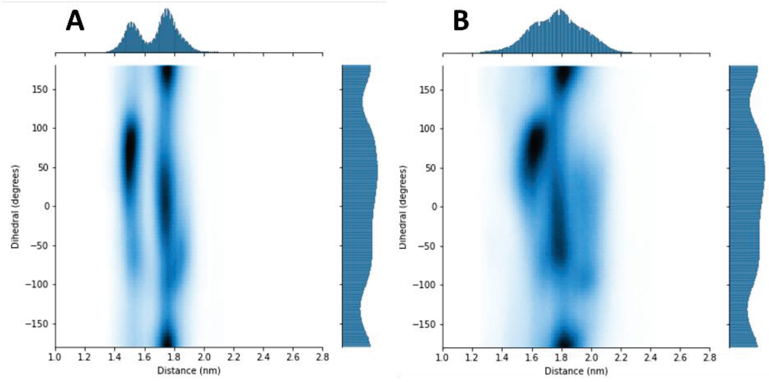

Figure S12: Joint distribution functions comparing the dihedral angle and distance of Trp_D calculated with respect to FAD using two different distance methods. Panel (a) depicts the centre-of-mass distances between the isoalloxazine ring of FAD (FLA1-5) and the entire tryptophan residue, providing an overall view of their spatial relationship. Panel (b) focuses on the distances between bead FLA2 of FAD and bead SC2 of tryptophan, which more closely represent the centres of spin density of the radical.

[11] Comparison of Distance and Dihedral Angles in Non-Flipped vs. Flipped Trajectories: Joint Distribution Plots

[a] TrpC

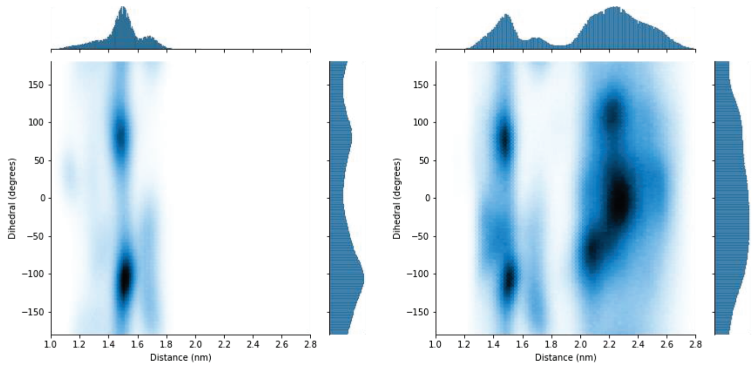

Figure S13: Joint distribution functions comparing the dihedral angle and distance of Trp_C calculated with respect to FAD; specifically calculating the distance from the bead FLA2 of FAD to the bead SC2 of tryptophan, corresponding closely to the centres of spin density on each radical. Panel (a) only considers trajectories where the Trp_C/Trp_D-rearrangment is absent. Panel (b) only considers trajectories where the Trp_C/Trp_D-rearrangment is presence.

[c] TrpD

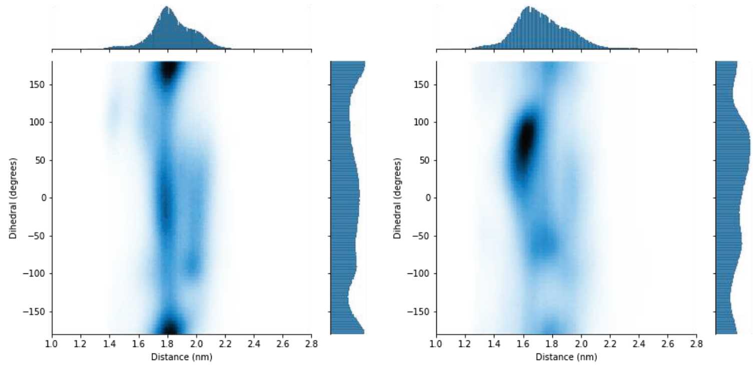

Figure S14: Figure S10: Joint distribution functions comparing the dihedral angle and distance of Trp_D calculated with respect to FAD; specifically calculating the distance from the bead FLA2 of FAD to the bead SC2 of tryptophan, corresponding closely to the centres of spin density on each radical. Panel (a) only considers trajectories where the Trp_C/Trp_D-rearrangment is absent. Panel (b) only considers trajectories where the Trp_C/Trp_D-rearrangment is presence.

[12] Comparison of dihedral angles between Trp_C and Trp_D: Joint Distribution plots

[a] Non-flipped vs. flipped trajectories

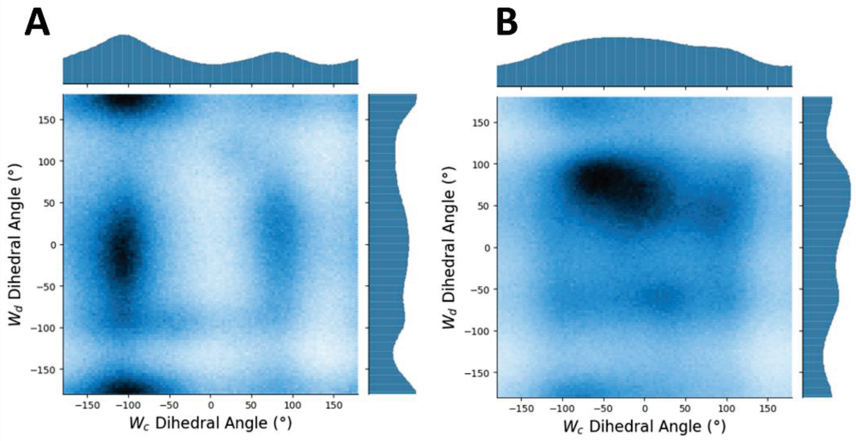

Figure S15: The dihedral angle of Trp_C vs Trp_D (labelled W_c and W_d, respectively). Panel (a) only considers trajectories where the Trp_C/Trp_D-rearrangment is absent. Panel (b) only considers trajectories where the Trp_C/Trp_D-rearrangment is presence.

[b] Initial microsecond vs. Final microsecond

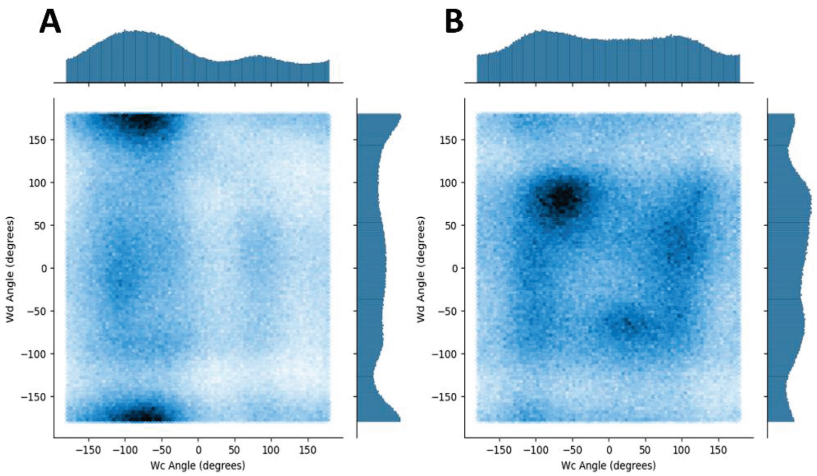

Figure S16: The dihedral angle of Trp_C vs Trp_D (labelled W_c and W_d, respectively). Panel (a) displays the initial microsecond (0-1 μs) of the simulation, while panel (b) represents the final microsecond (19-20 μs).

[c] Visual illustration of the closed and open confirmations obtained via blender.

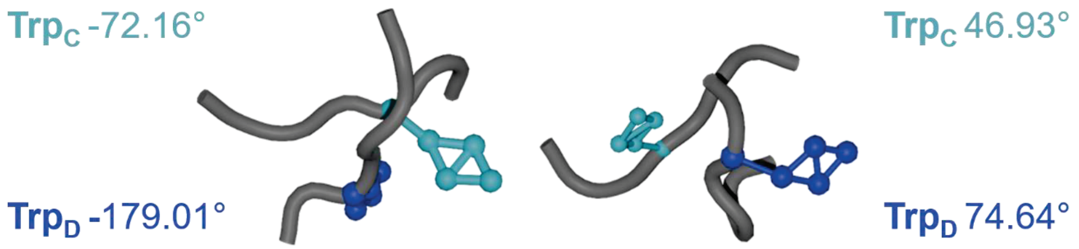

Figure S17: The visual representation of the (a) absence of Trp_C/Trp_D-rearrangment and (b) the presenc of Trp_C/Trp_D-rearrangment. We can see that, initially, the two tryptophan are close together, but are separated through the Trp_C/Trp_D-rearrangment. The displayed dihedral angles for each of these configurations are as follows: for the non-flipped {Trp_C: -72.16° , Trp_D: -179.01°} and flipped {Trp_C: 46.93° , Trp_D: -74.64°}.

[13] Average RMSD Comparison of Specific Regions in absence and presence of the Trp_C/Trp_D-rearrangment

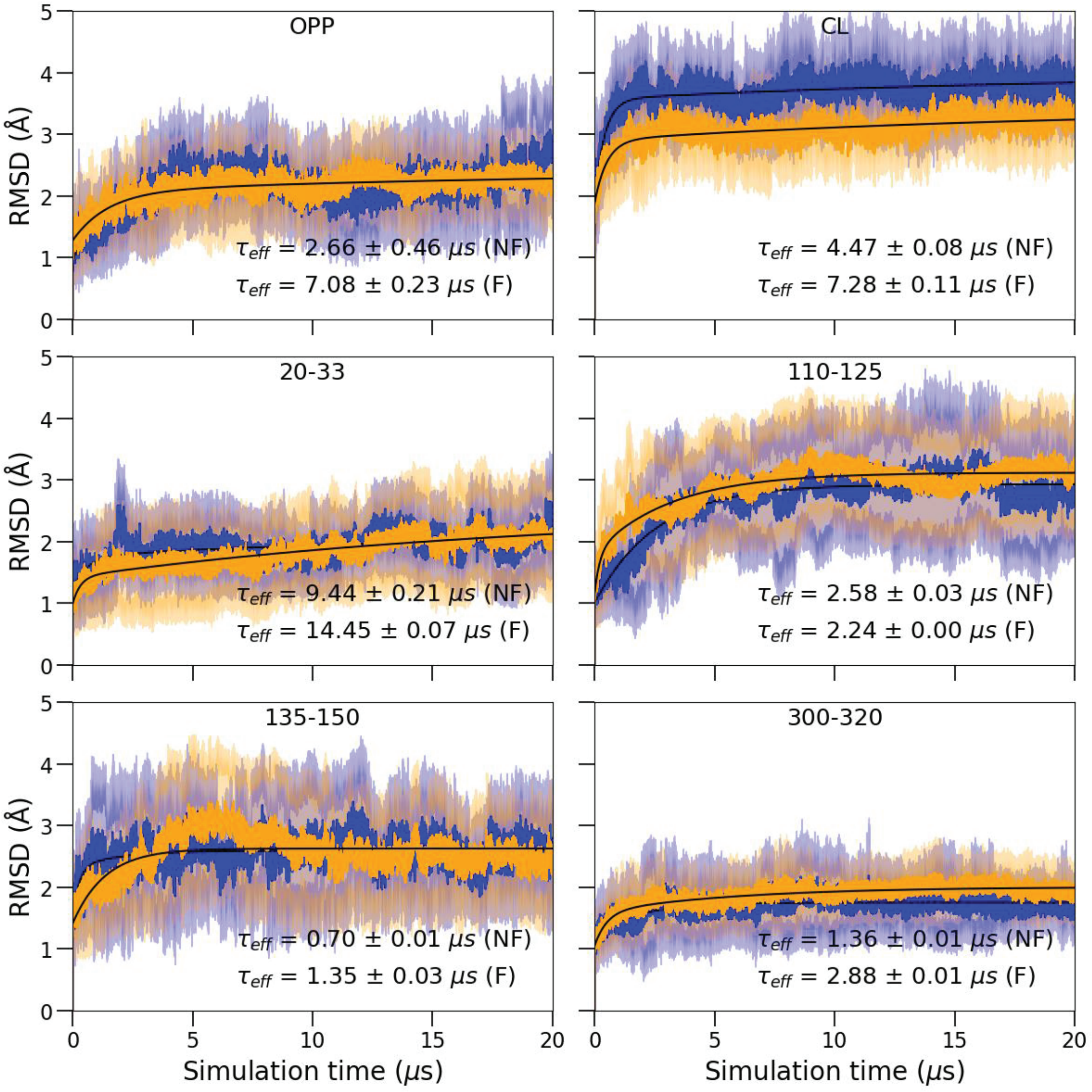

Figure S18: The average RMSD and standard deviation of additional regions of the ClCry4 protein comparing the systems with (orange) and without (blue) the Trp_C/Trp_D rearrangement. The bold colours represent the mean RMSD, while transparent colours depict the standard deviation. Twelve of the trajectories exhibited the Trp_C/Trp_D rearrangement, and the remaining eight did not.

[14] Similarity Measures in absence and presence of Trp_C/Trp_D-rearrangment

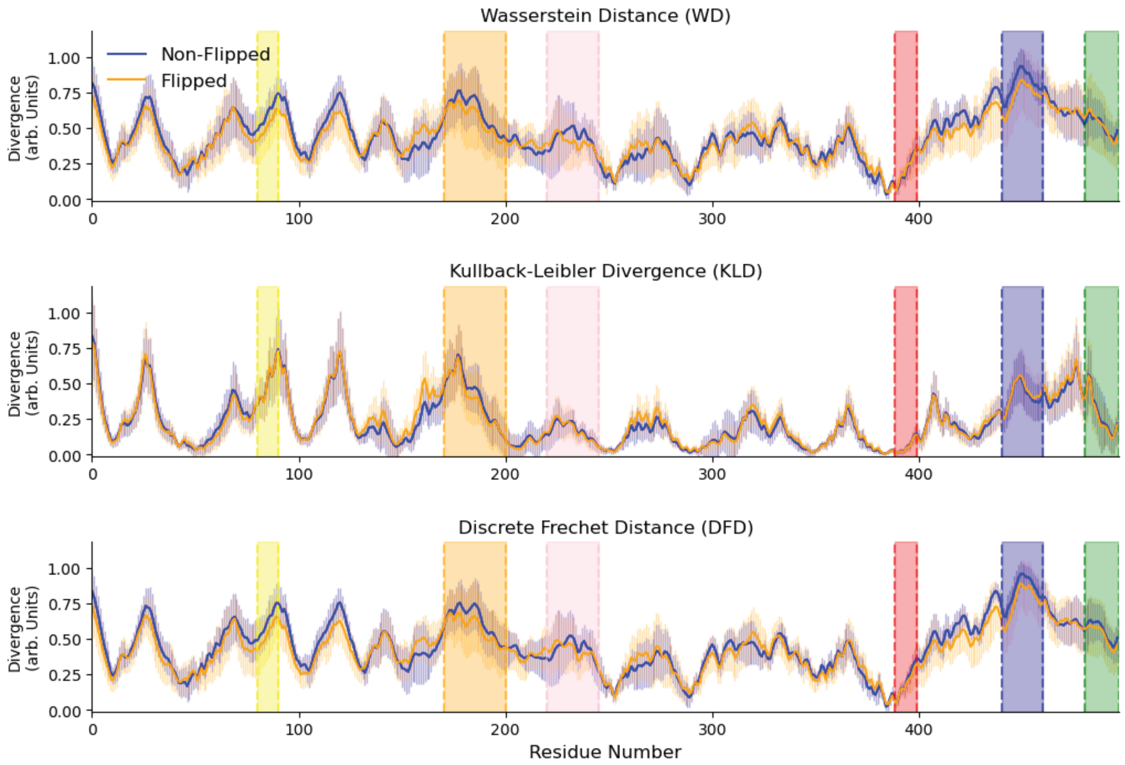

Figure S19: Comparison of the average divergence in (a) Wasserstein Distance (WD), (b) Kullback-Leibler Divergence (KLD), and (c) Discrete Fréchet Distance (DFD) for trajectories involving a change in the Trp_C distance from the FAD, corresponding to a side chain flip. The divergence is shown for non-flipped systems (blue) and flipped systems (orange) across protein residues. Noticeably larger divergence is observed at the end of the protein, particularly in the EEE and C-terminal regions, with higher standard deviation around these areas. For all three similarity measures, we see very similar divergence across the residues.

[15] Network Communities identified via label propagation function

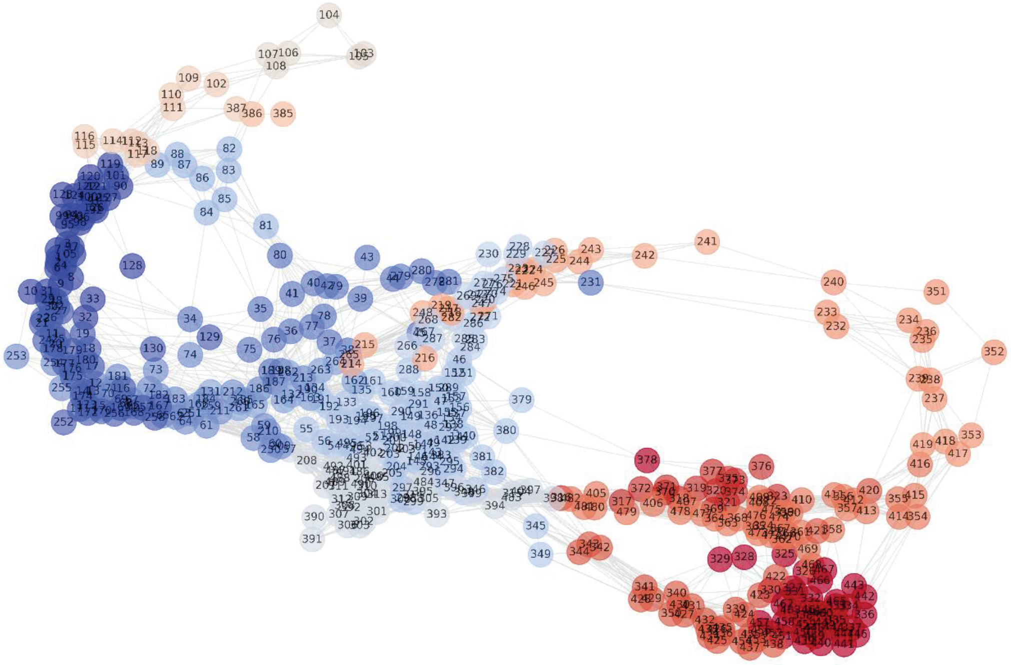

Figure S20: Graph resulting from the correlation matrix of the normalised Kullback-Leibler divergence for all trajectories. The threshold of 0.86 applied to filter the correlation matrix, highlighting only the strong correlations among the trajectories. Colours corresponding to different communities identified via label propagation using NetworkX. A total of eighteen communities were identified.

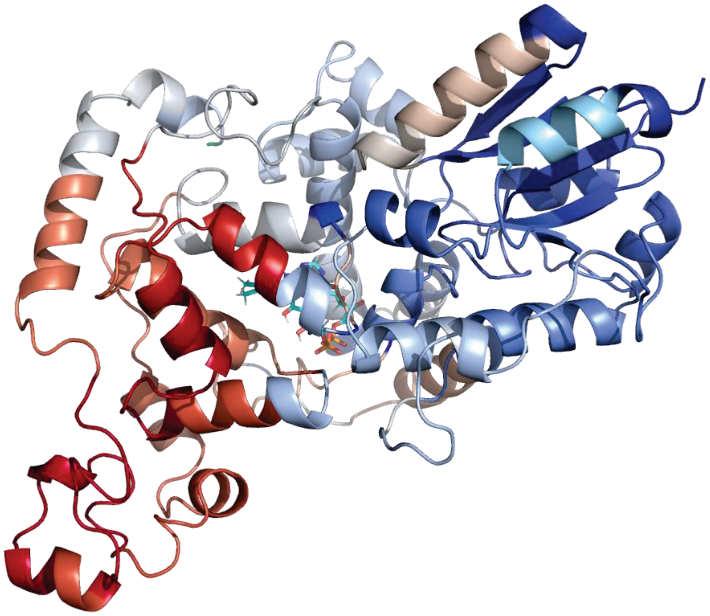

Figure S21: The eighteen communities visualised on the protein using the same colours as depicted in Fig. S20.

[16] Network Centralities and Hubs

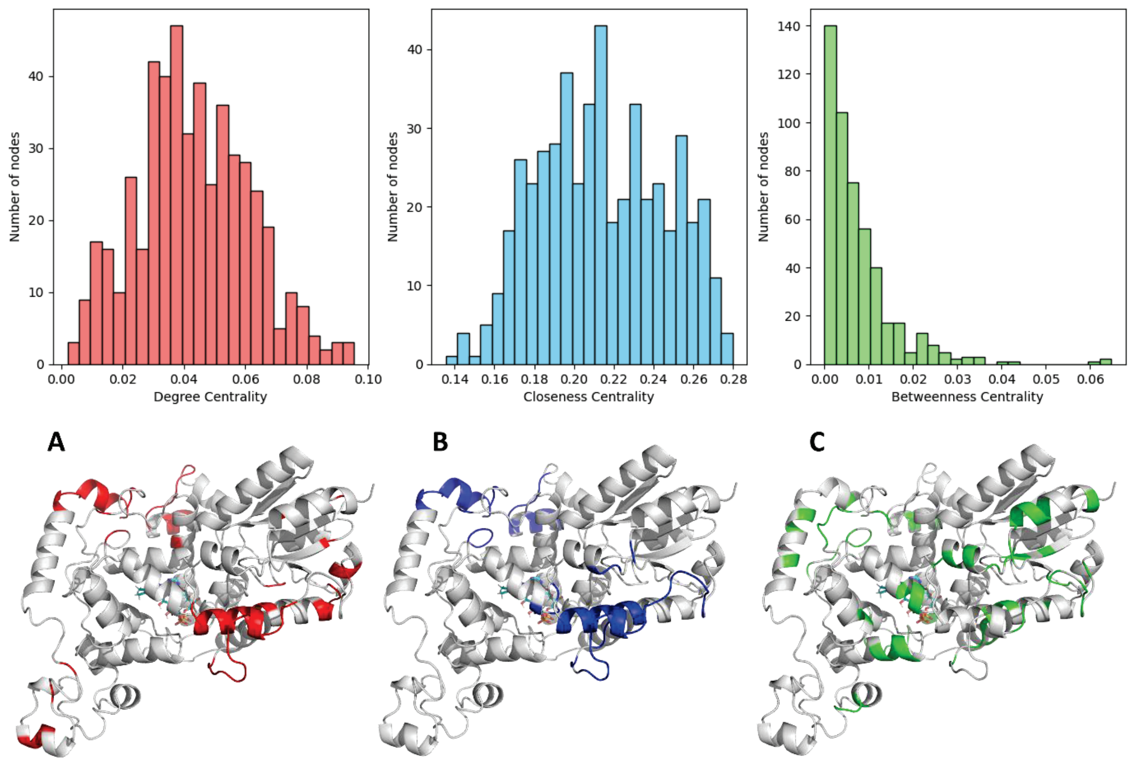

Figure S22: The histograms displayed the degree, closeness and betweenness centralities calculated from the correlation matrix of the normalised Kullback-Leibler divergence for all trajectories. The threshold of 0.86 applied to filter the correlation matrix, highlighting only the strong correlations among the trajectories. The top 15% of each centrality is visualised on the protein structure. (A) Degree centrality (red), (B) closeness centrality (blue), and (C) betweeness centrality (green). Twelve common residues across all three centralities include: {56, 59, 149, 153, 155, 192, 193, 206, 313, 400, 493, 496}s.

Within the top 15% in degree centrality, three of the regions {CL: 13, EEE: 4, CT; 11} had residues identified. Within the top 15% in closeness centrality: two of the regions {CL: 12, CT: 16} had residues identified. Within the top 15% in betweeness centrality: four of the regions {OPP: 6, PBL: 5, CL: 7, CT: 5} had residues identified.
