# Supplementary material for: Cryptochrome magnetoreception: Time course of photoactivation from non-equilibrium coarse-grained molecular dynamics
Source: Comput Struct Biotechnol J. 2024 Nov 10;26:58–69. doi: 10.1016/j.csbj.2024.11.001 (PMC11725172; doi:10.1016/j.csbj.2024.11.001)
Supplement: Supplementary file 2 — Analysis codes and cg-MD simulation inputs (including initial molecular structures, parameters and topology files) and outputs (final molecular structures) of the 20 replicas. [file mmc2.zip › cg-md/data/readme.docx]

MD Trajecotry Files:

Mus_XX.gro file: is the pdb for the first frame of that respective trajectory

Production.gro: is the pdb of the final frame of that respective trajectory

Cg_cry.top: The topology file, containing the molecular structure and interaction details.

Production.mdp: The parameter file that defines the simulation settings for the production phase.
