# Supplementary material for: Cryptochrome magnetoreception: Time course of photoactivation from non-equilibrium coarse-grained molecular dynamics
Source: Comput Struct Biotechnol J. 2024 Nov 10;26:58–69. doi: 10.1016/j.csbj.2024.11.001 (PMC11725172; doi:10.1016/j.csbj.2024.11.001)
Supplement: Supplementary file 2 — Analysis codes and cg-MD simulation inputs (including initial molecular structures, parameters and topology files) and outputs (final molecular structures) of the 20 replicas. [file mmc2.zip › code/readme.docx]

**Code Descriptions:**

Code 01:

This code calculates the Root Mean Square Deviation (RMSD) for a protein, focusing on the entire protein as well as nine specific regions using the MDAnalysis package [1, 2]. The RMSD calculation is used to assess the structural differences between various states of the protein. This data is included in both the main manuscript and supporting information.

Code 02:

This code deals with similarity measures calculated using the SiMBols toolkit [3]. It specifically computes three types of distances: Wasserstein Distance (WD), Kullback-Leibler Distance (KLD), and Discrete Fréchet Distance (DFD). The code first processes and converts trajectory data into numpy files, aligns these trajectories to the backbone atoms of a specific protein state (dark state), and then calculates the similarity measures for each trajectory.

Code 03:

This code creates a correlation matrix using the KLD data obtained from Code 02. It then uses NetworkX [4], a Python library for graph analysis, to visualize the correlations as a network graph, showing the connections between different protein regions based on their similarity.

Code 04:

This code involves fitting functions and parameters used to model the RMSD data for various regions of the protein. Additionally, it analyses the RMSD data considering the presence or absence of specific protein re-arrangements (TrpC/TrpD), allowing for the analysis of how these re-arrangements affect the protein's structure.

1. Michaud-Agrawal, N., et al., *MDAnalysis: A toolkit for the analysis of molecular dynamics simulations.* Journal of Computational Chemistry, 2011. **32**(10): p. 2319-2327.

2. Gowers, R.J., et al., *MDAnalysis: A Python Package for the Rapid Analysis of Molecular Dynamics Simulations*, in *Conference: PROC. OF THE 15th PYTHON IN SCIENCE CONF. (SCIPY 2016) ; 2016-07-11 - 2016-07-11 ;*. 2019: United States. p. Medium: ED; Size: 98.

3. Schuhmann, F., et al., *Across atoms to crossing continents: Application of similarity measures to biological location data.* PLOS ONE, 2023. **18**(5): p. e0284736.

4. Hagberg, A., P. Swart, and D. Chult, *Exploring Network Structure, Dynamics, and Function Using NetworkX*. 2008.
